# Supplementary material for: The hybrid lipoplex induces cytoskeletal rearrangement via autophagy/RhoA signaling pathway for enhanced anticancer gene therapy
Source: Nat Commun. 2025 Jan 2;16:339. doi: 10.1038/s41467-024-55727-4 (PMC11696071; doi:10.1038/s41467-024-55727-4)
Supplement: Supplementary file 1 — Supplementary Information [file 41467_2024_55727_MOESM1_ESM.pdf]

Supporting Information

The hybrid lipoplex induces cytoskeletal rearrangement via autophagy/RhoA signaling pathway for enhanced anticancer gene therapy

Xueyi Hu[†, 1], *Yichun Wang*[†, 1], *Ruohan Wang* [1], *Yiyao Pu* [1], *Rongrong Jin*[*, 1], *Yu Nie*[*, 1], *Xintao Shuai*[2]

[†] Equal contribution

1 National Engineering Research Center for Biomaterials, College of Biomedical Engineering, Sichuan University, Chengdu 610064, P. R. China

2 Nanomedicine Research Center, The Third Affiliated Hospital of Sun Yat-sen University, Guangzhou 510630, P. R. China

*Corresponding authors, Email: nie_yu@scu.edu.cn (Yu Nie); jinrr2015@scu.edu.cn (Rongrong Jin)

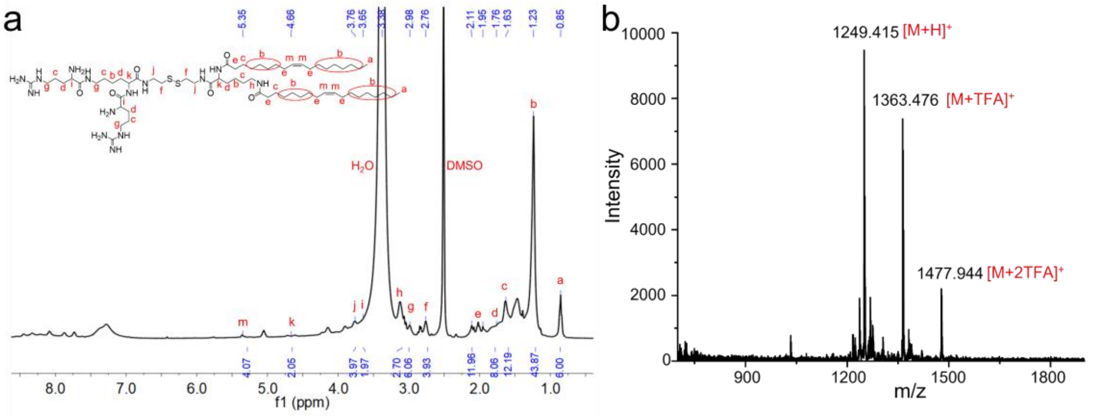

Supplementary Figure 1. Characterization of RLS. a ^{1}H NMR spectra and **b** matrix-assisted laser desorption ionization time-of-flight mass spectrometry of RLS. Representative of 3 independent experiments with similar results. Source data are provided as a Source Data file.

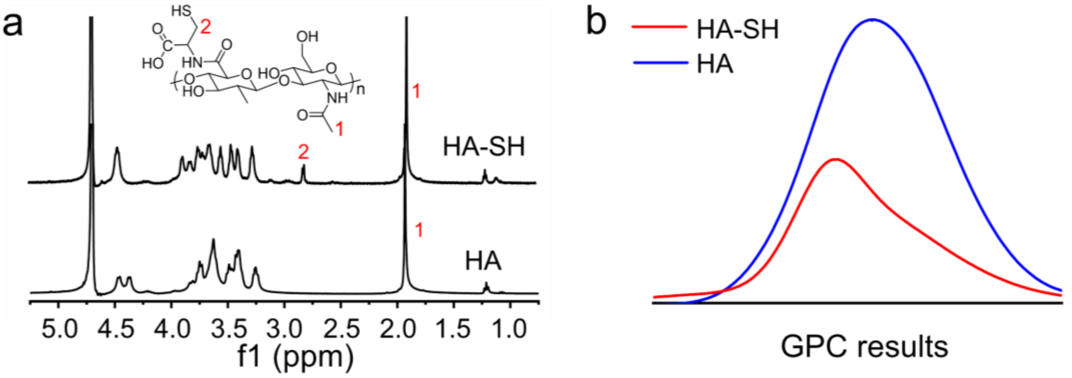

Supplementary Figure 2. Characterization of HA-SH(HS). a ^{1}H NMR spectra of hyaluronic acid (HA) and thiol conjugated HA (HS) in D_2O. **b** The GPC results of HA and HS. The number-average molecular of HA was 36228 with a polydispersity of 2.53, and the HA-SH was 47943 with a polydispersity of 2.34. Representative of 3 independent experiments with similar results. Source data are provided as a Source Data file.

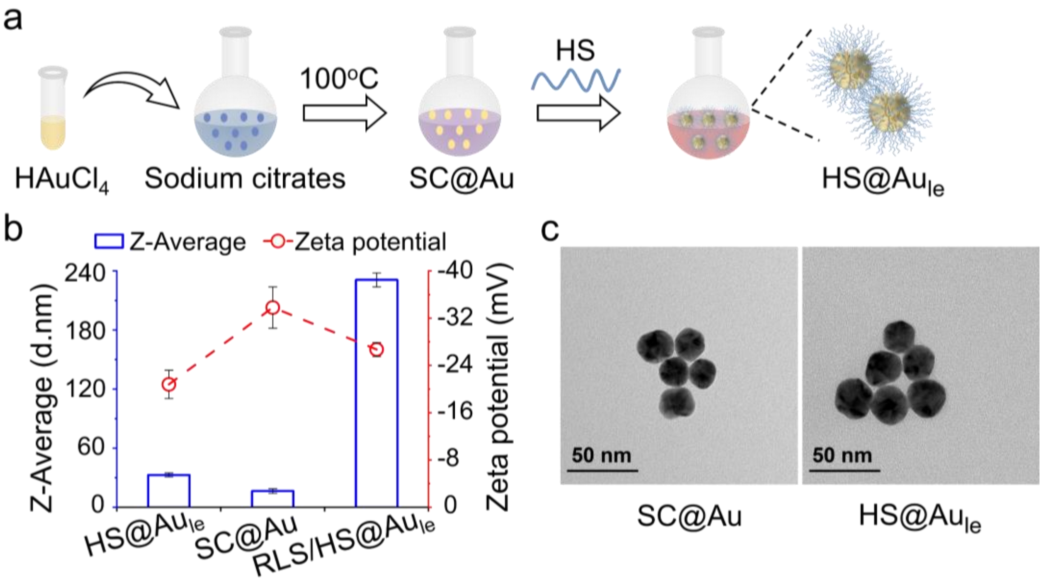

Supplementary Figure 3. Characterization of gold nanoparticles. a Synthetic scheme of SC@Au and HS@Au$_{le}$. **b** Size distribution and ζ potential of HS@Au$_{le}$, SC@Au and RLS/HS@Au$_{le}$ gene lipoplexes. n = 3 independent experimental units. The data are mean ± SD. **c** The representative TEM image of SC@Au, and HS@Au$_{le}$. n = 3 independent experimental units with similar results. The scale bar is 50 nm. Source data are provided as a Source Data file.

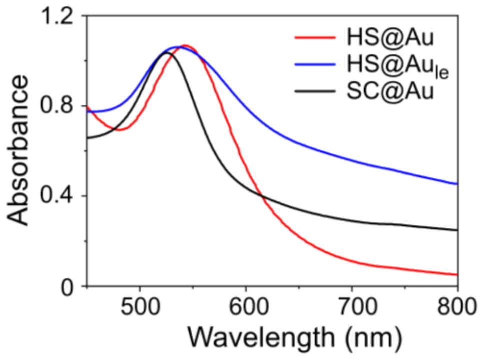

Supplementary Figure 4. UV-vis absorption spectra of HS@Au, HS@Au$_{le}$, and SC@Au. Representative of 3 independent experiments with similar results. Source data are provided as a Source Data file.

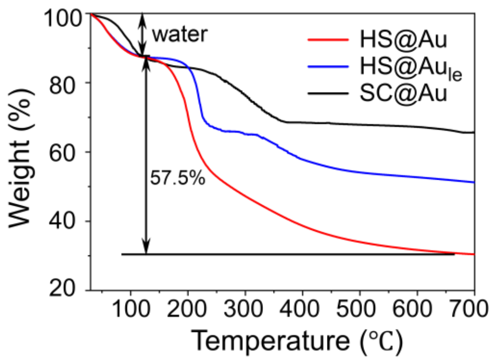

Supplementary Figure 5. TGA curves of HS@Au, HS@Au$_{le}$, and SC@Au. Representative of 3 independent experiments with similar results. Source data are provided as a Source Data file.

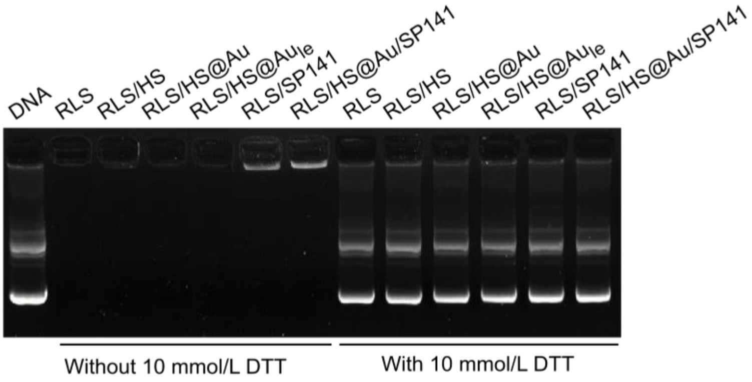

Supplementary Figure 6. Release behavior of DNA in various gene lipoplexes (RLS, RLS/HS, RLS/HS@Au, RLS/HS@Au$_{le}$, RLS/SP141, and RLS/HS@Au/SP41) with or without reductive agent (DTT, 10 mmol/L) after 2 h incubation at 37 °C. Representative of 3 independent experiments with similar results.

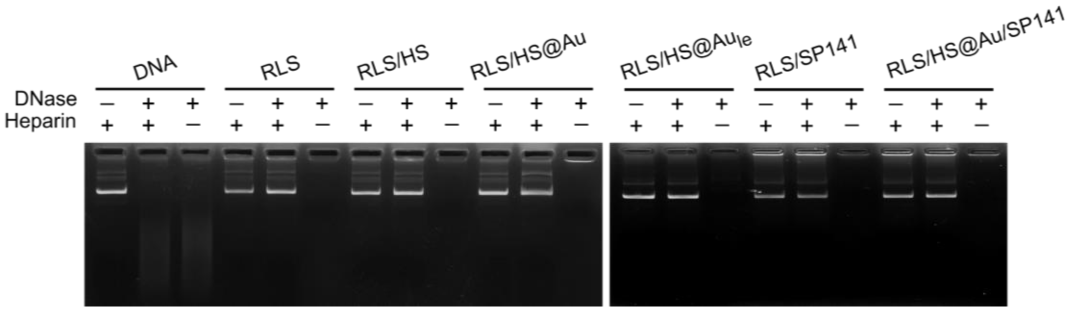

Supplementary Figure 7. Resistance capacity of various lipoplexes (RLS, RLS/HS, RLS/HS@Au, RLS/HS@Au$_{le}$, RLS/SP141, RLS/HS@Au/SP141) against DNase indicated by agarose gel electrophoresis. Different lipoplexes were incubated with DNase (200 U/mL) and/or heparin (4 mg/mL). Representative of 3 independent experiments with similar results.

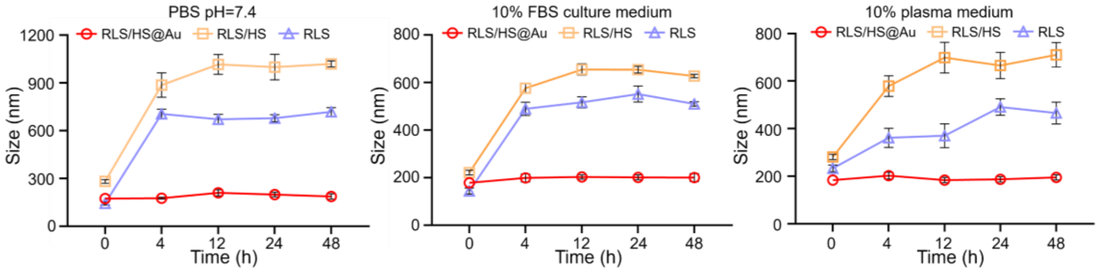

Supplementary Figure 8. Size changes of different nanoparticles (RLS, RLS/HS, and RLS/HS@Au) by DLS measurement in the indicated conditions, including PBS buffer (pH 7.4), culture medium with 10% FBS, and 10% plasma. The data are mean ± SD. n = 3 independent experimental units. Source data are provided as a Source Data file.

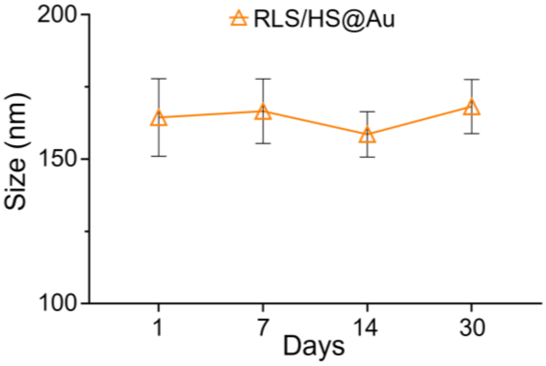

Supplementary Figure 9. Size distribution of RLS/HS@Au in one month. The data are mean ± SD. n = 3 independent experimental units. Source data are provided as a Source Data file.

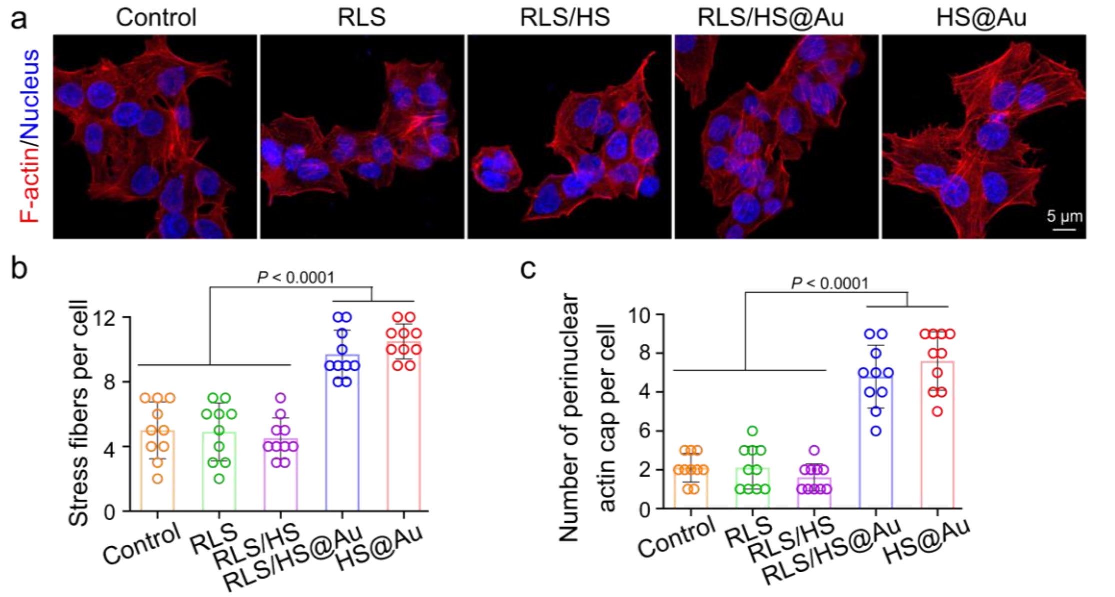

Supplementary Figure 10. Cytoskeleton observation after different lipoplexes

treatment. a Representative image of cells stained with Rhodamine (red) conjugated phalloidin and DAPI (blue) after treatment with various gene lipoplexes and HS@Au for 24 h. n = 3 independent experiments cell lines. The scale bar is 5 μm. **b** Quantification of the stress fiber. n = 10 cells from three independent experimental cell lines. **c** Quantification of the perinuclear actin cap per cell. n = 10 cells from 3 independent experimental cell lines. One-way ANOVA with Tukey's post hoc test was used for the comparisons in (**b**) and (**c**). The data in (**b**, **c**) are mean ± SD. p values < 0.05 were considered statistically significant. Source data are provided as a Source Data file.

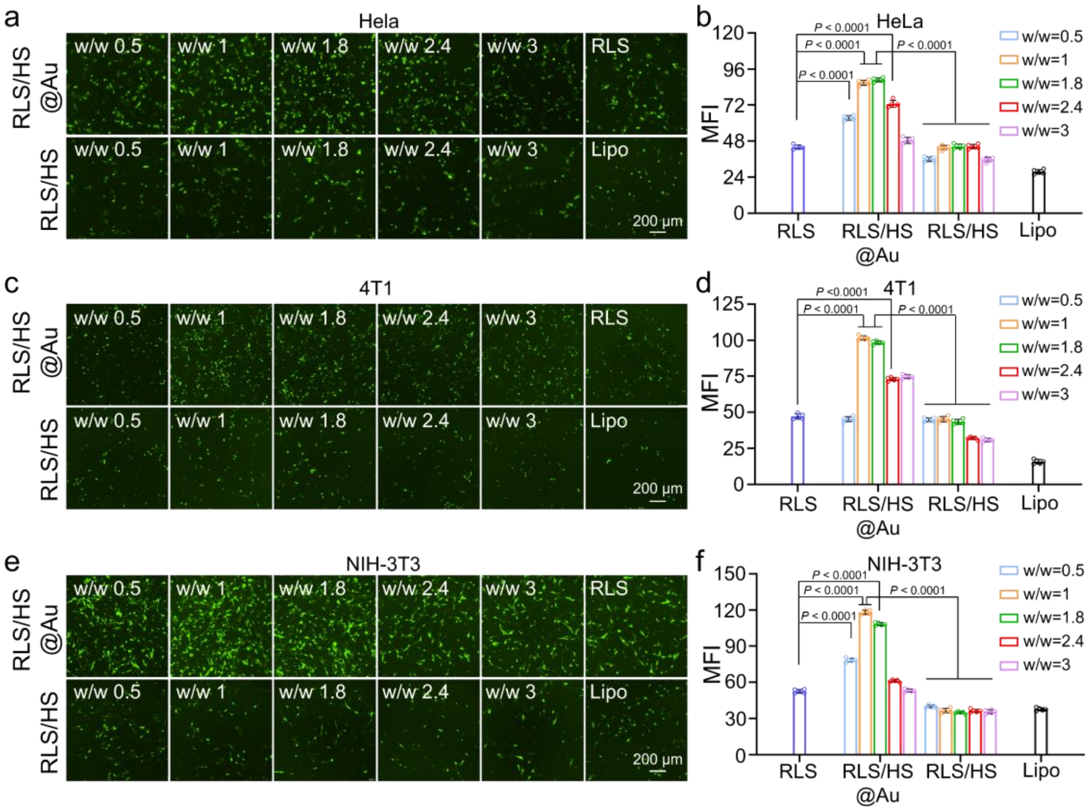

Supplementary Figure 11. In vitro transfection of various gene lipoplexes on different cells. Representative fluorescence microscopy images of EGFP transfected HeLa (**a**), 4T1(**c**), and NIH-3T3 (**e**) cells. n = 3 independent experimental cell lines with similar results. The scale bar is 200 μm. The semi-quantitative analysis of the mean fluorescence intensity (MFI) of HeLa (**b**), 4T1 (**d**), and NIH-3T3 (**f**) cells using ImageJ software. n = 5 fields from 3 independent experimental cell lines. Two-sided unpaired Student's t test was used for the comparisons in (**b**), (**d**) and (**f**). The data in (**b**, **d**, and **f**) are mean ± SD. p values < 0.05 were considered statistically significant. Source data are provided as a Source Data file.

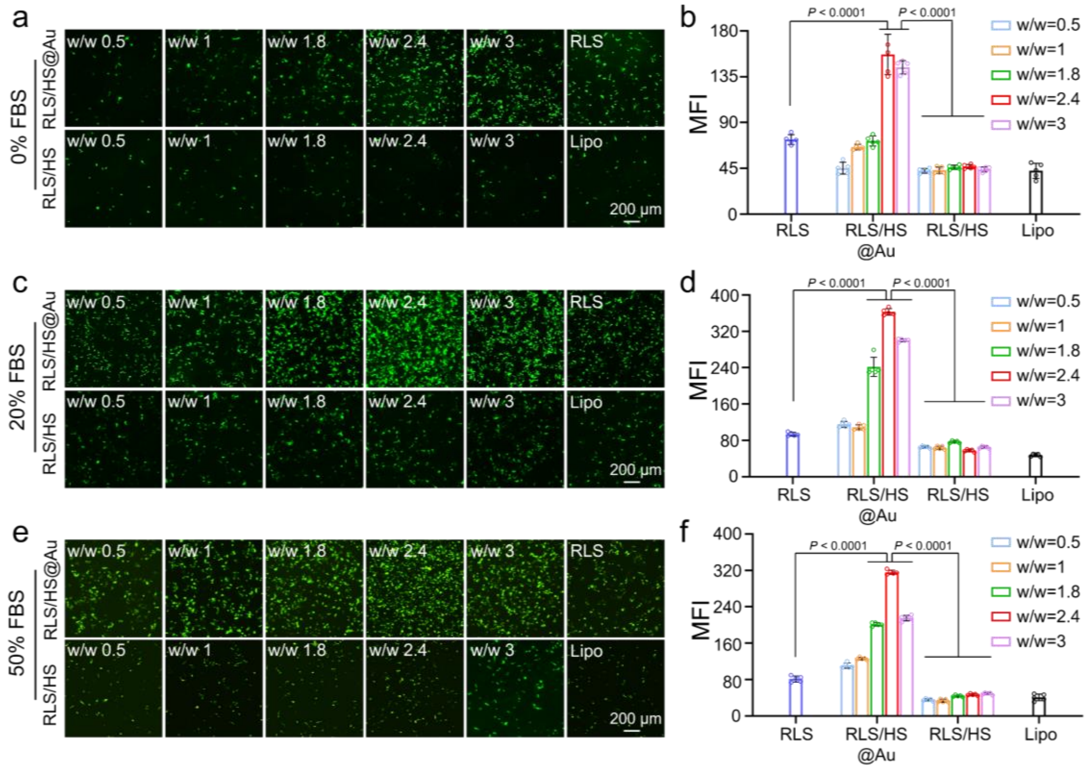

Supplementary figure 12. In vitro transfection of RLS, RLS/HS, and RLS/HS@Au lipoplexes on HepG2 cells in culture medium with different serum concentrations. Representative fluorescence microscopy images of the pEGFP transfected cells in 0% (**a**), 20% (**c**), and 50% (**e**) serum. n = 3 independent experimental cell lines with similar results. The scale bar is 200 μm. The corresponding semi-quantitative analysis of the mean fluorescence MFI (**b**, **d**, and **f**) was performed by ImageJ software. n = 5 fields from 3 independent experimental cell lines). Two-sided unpaired Student's t test was used for the comparisons in (**b**), (**d**), and (**f**). The data in (**b**, **d**, and **f**) are mean ± SD. p values < 0.05 were considered statistically significant. Source data are provided as a Source Data file.

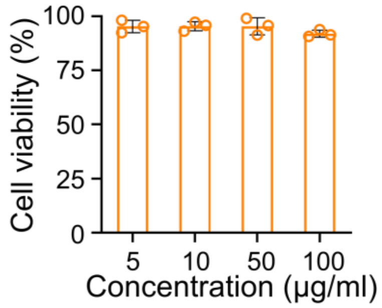

Supplementary Figure 13. Cell viability of HS@Au on HepG2 cells. The data are mean ± SD. n = 3 independent experimental cell lines. Source data are provided as a Source Data file.

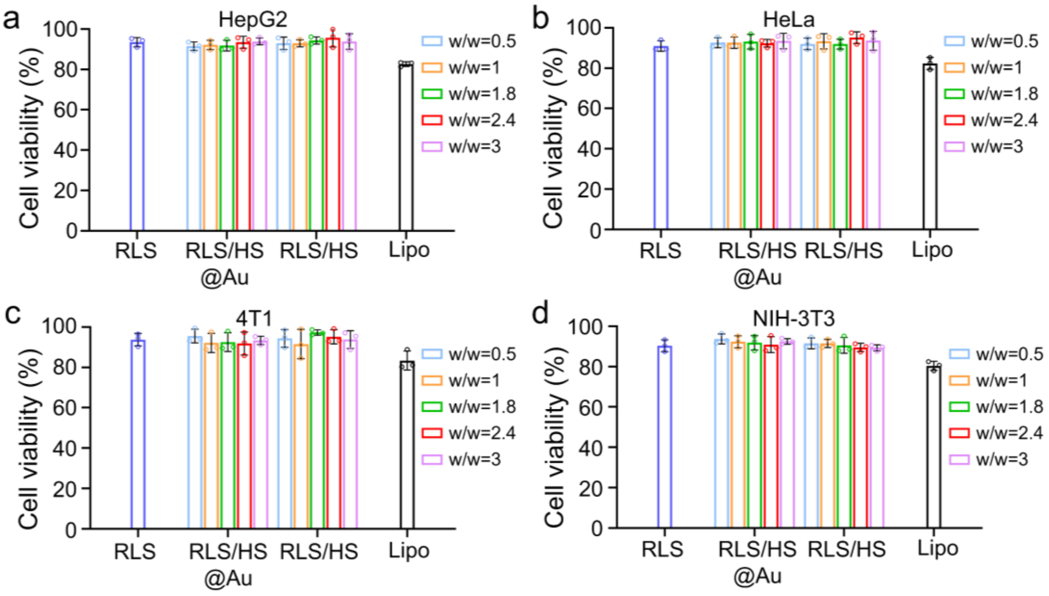

Supplementary Figure 14. Cytotoxicity assessment of hybrid lipoplexes on multiple cell lines. Cellular viability of various gene lipoplexes in HepG2 (**a**), HeLa (**b**), 4T1 (**c**), and NIH-3T3 (**d**) cells with different Au to pDNA ratios (w/w). The N/P ratio was fixed at 20 and the final concentration of pEGFP was 2 μg/mL. The data are mean ± SD. n = 3 independent experimental cell lines. Source data are provided as a Source Data file.

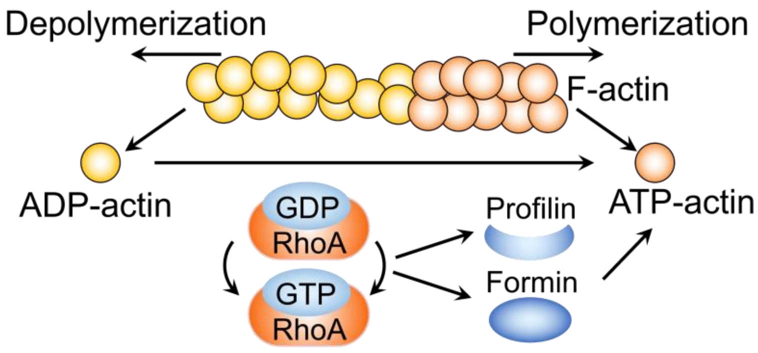

Supplementary Figure 15. Schematic illustration of the GTP-RhoA associated dynamic rearrangement of F-actin/cytoskeleton in HepG2 cells.

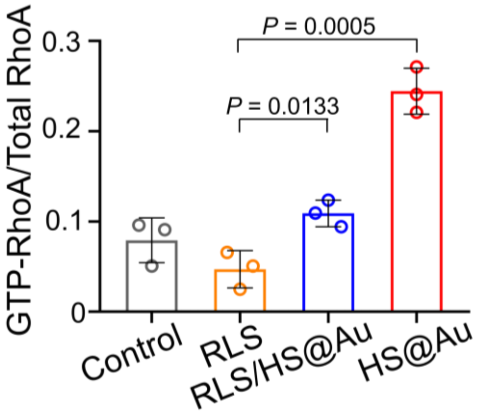

Supplementary Figure 16. The expression levels of GTP-RhoA quantified via the gray intensity analysis (normalized to the total RhoA). n = 3 independent experimental cell lines. Two-sided unpaired Student's t test was used for the comparisons. The data are mean ± SD. *p* values < 0.05 were considered statistically significant. Source data are provided as a Source Data file.

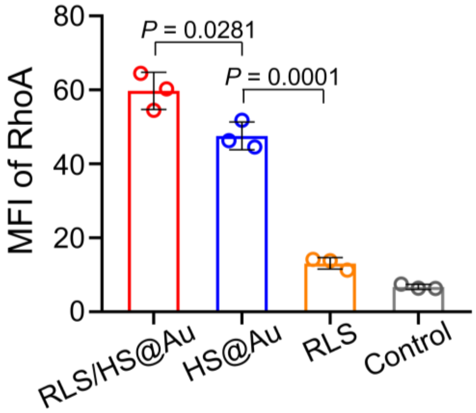

Supplementary Figure 17. The mean fluorescence intensity of RhoA quantified by the ImageJ software. n = 3 fields from 3 independent experimental cell lines. Two-sided unpaired Student's t test was used for the comparisons. The data are mean ± SD. *p* values < 0.05 were considered statistically significant. Source data are provided as a Source Data file.

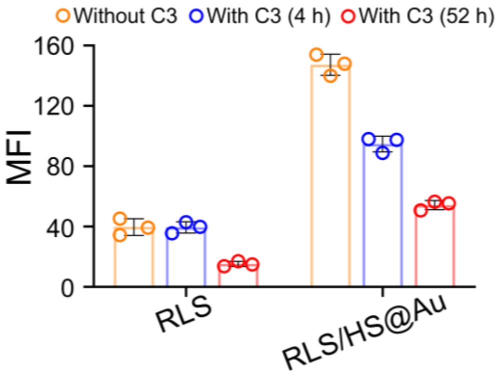

Supplementary Figure 18. Mean fluorescence intensity (MFI) of HepG2 cells after transfected with various gene lipoplexes in the absence or presence of C3 transferase. n = 3 fields from 3 independent experimental cell lines. The data are mean ± SD. Source data are provided as a Source Data file.

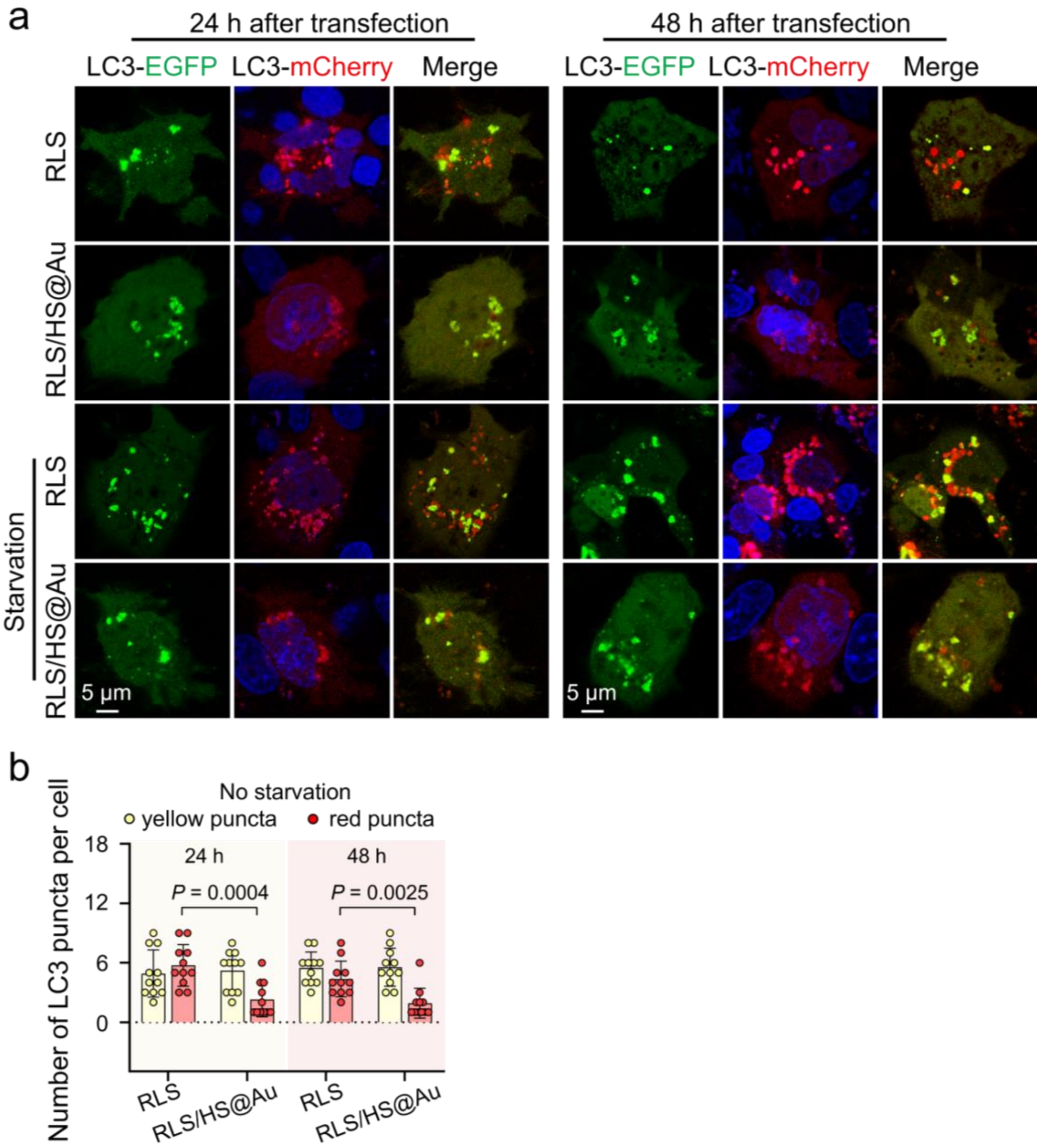

Supplementary Figure 19. The monitoring of autophagic flux. a Representative confocal image of HepG2 cells expressing LC3-EGFP-mCherry after treatment with various gene lipoplexes for 24 and 48 h. In the starvation conditions, HepG2 cells were cultured in EBSS buffer for 5 h after lipoplexes treatment. Green tunnel: LC3-EGFP; Red tunnel: LC3-mCherry; Yellow tunnel: the merge of EGFP and mCherry, Blue: DAPI. n = 3 independent experimental cell lines with similar results. The scale bar is 5 μm. **b** Mean number of yellow puncta and red puncta. n = 11 cells from 3 independent experimental cell lines. Two-sided unpaired Student's t test was used for the comparisons. The data are mean ± SD. p values < 0.05 were considered statistically significant. Source data are provided as a Source Data file.

Control

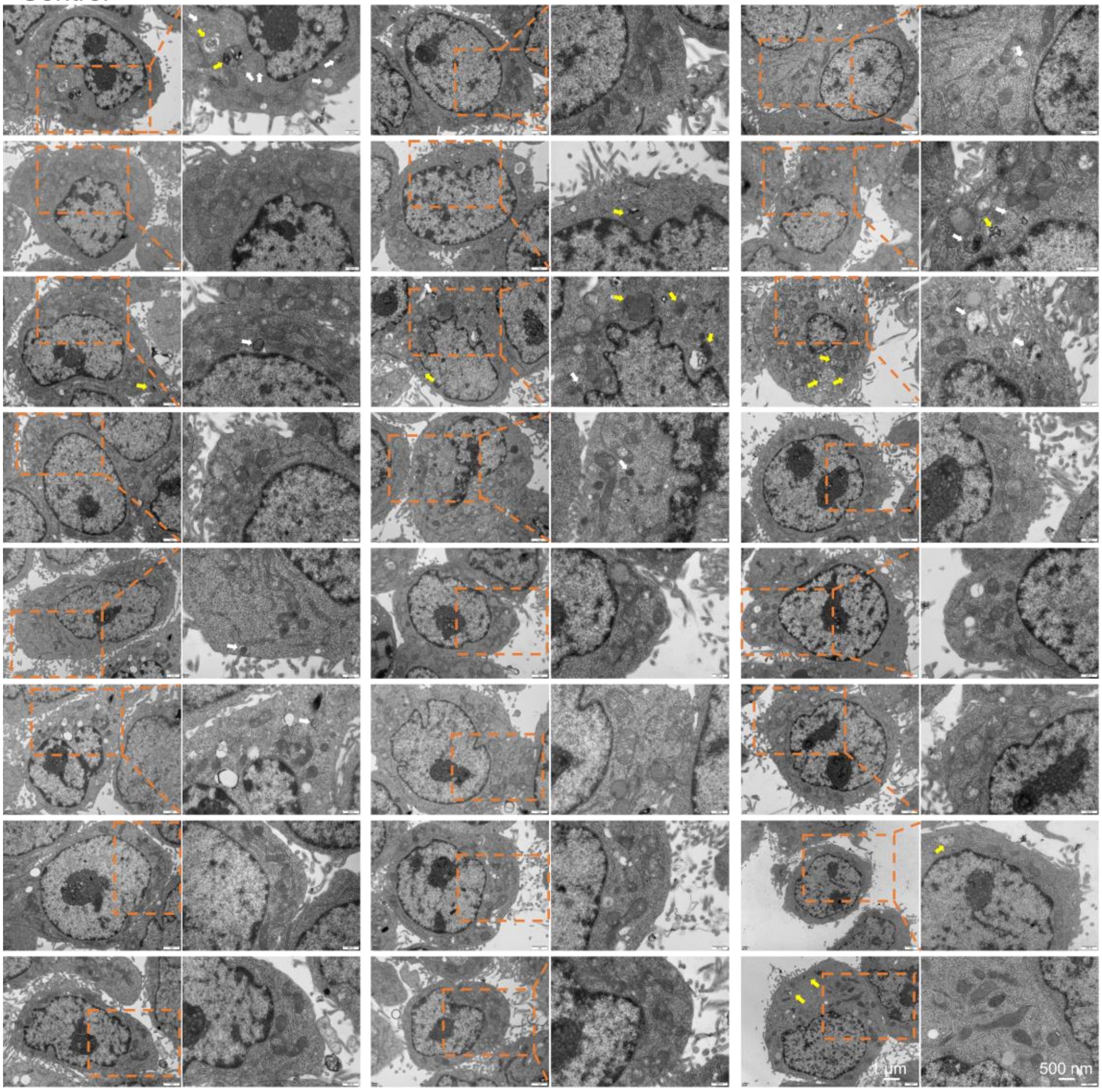

RLS/HS@Au

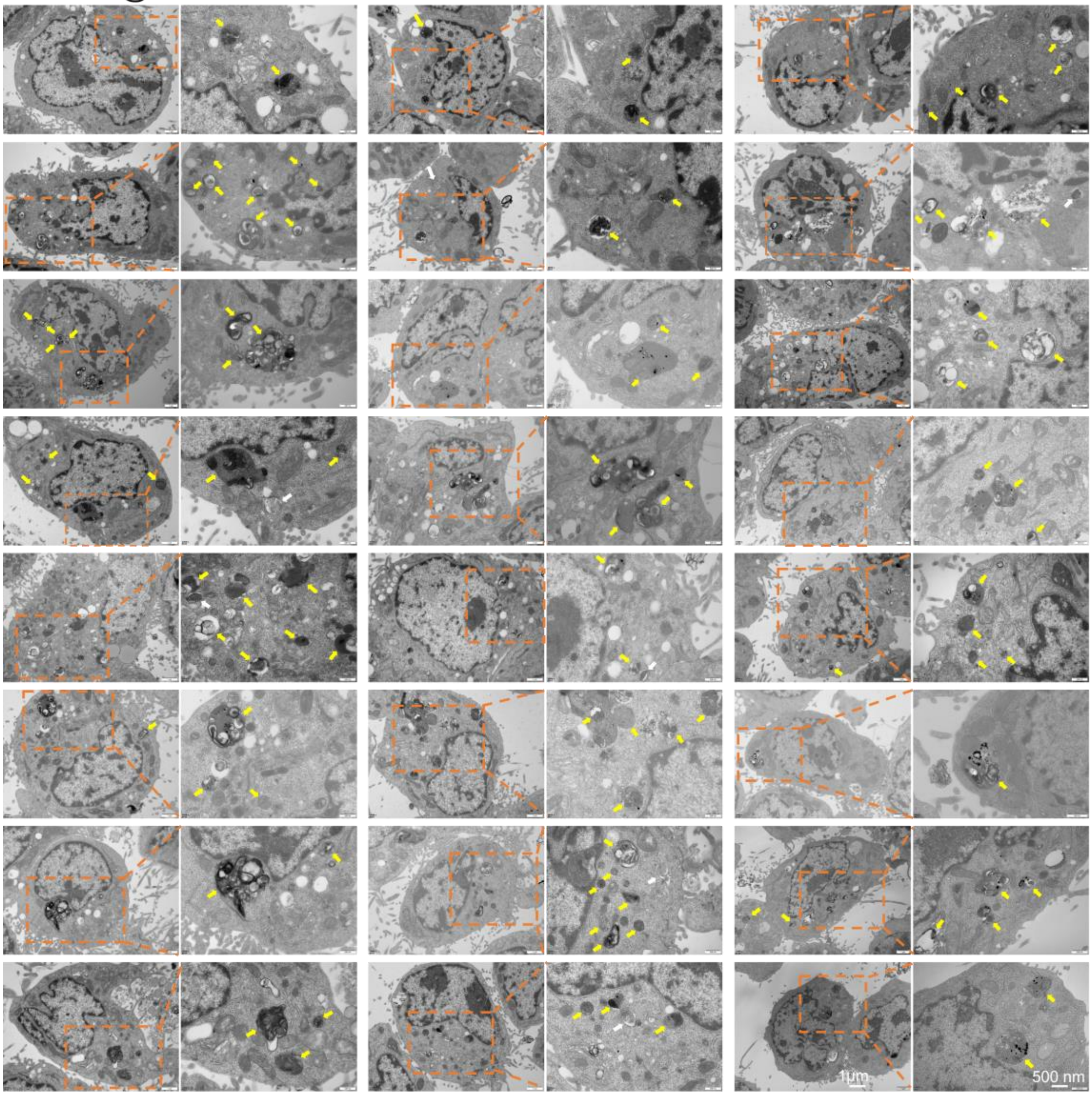

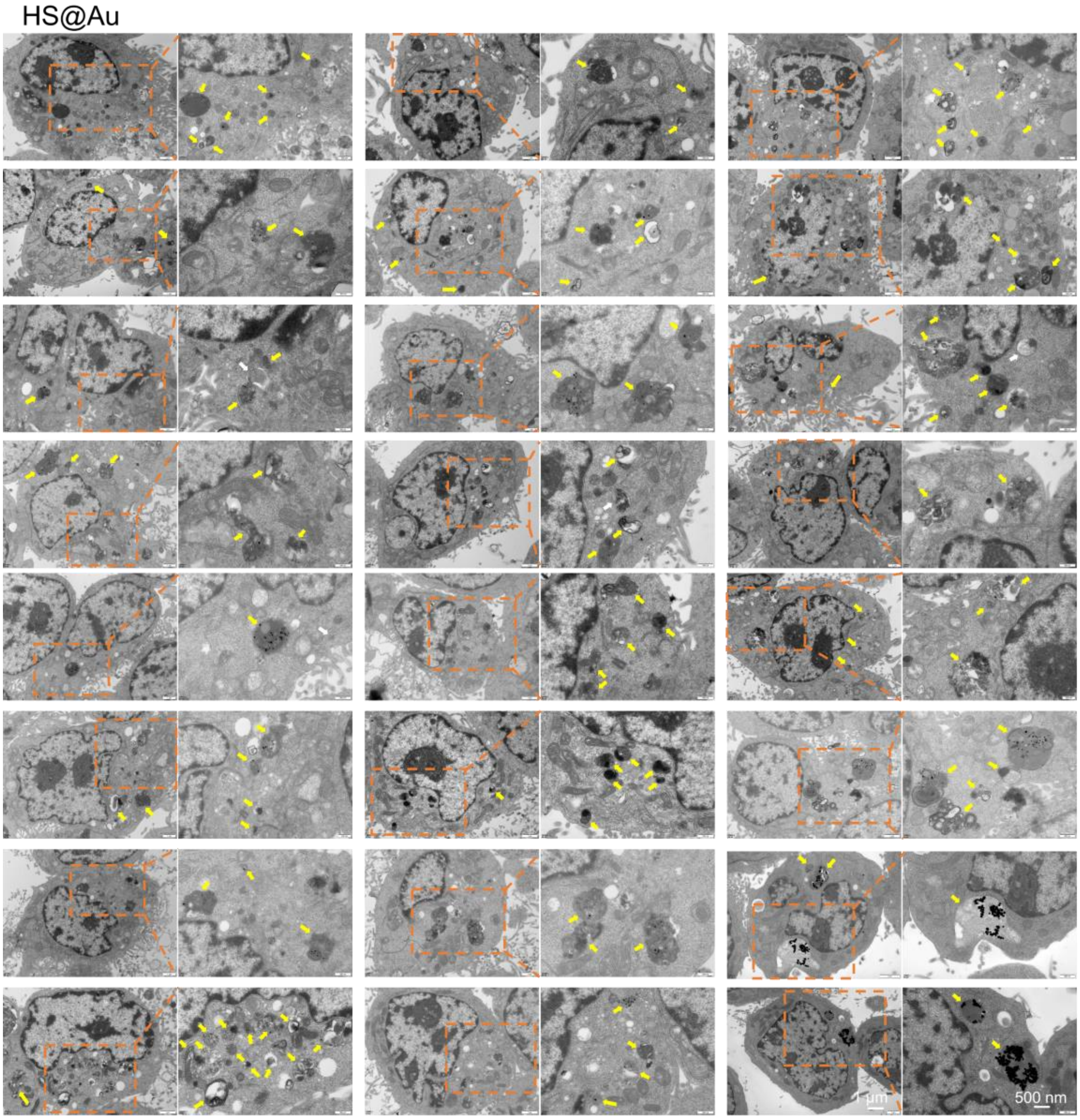

Supplementary Figure 20. Bio-TEM observation of autophagic flux induced by RLS/HS@Au at DNA of 1 μg/mL or free HS@Au (50 μg Au/mL) on HepG2 cells. The white and yellow arrows indicated autophagosomes and autolysosomes, respectively. n = 25 cells from 3 independent experimental cell lines. The scale bars are 1 μm and 500 nm, respectively.

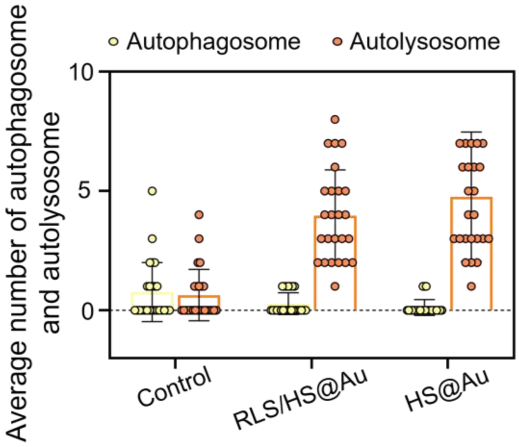

Supplementary Figure 21. The average number of autophagosomes and autolysosomes calculated from 25 cells in each group. n = 25 cells from 3 independent experimental cell lines. The data are mean ± SD. Source data are provided as a Source Data file.

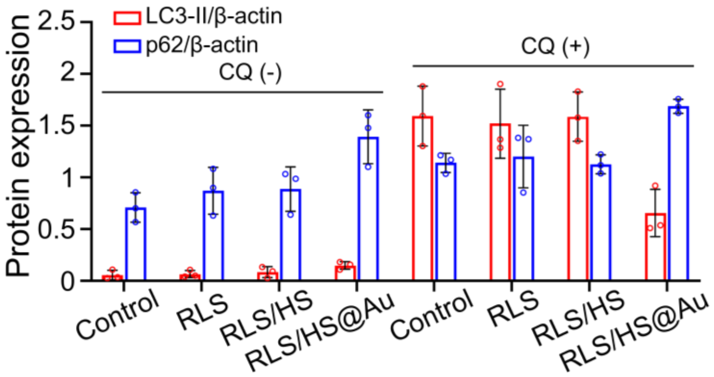

Supplementary Figure 22. The expression levels of LC3-II and p62 quantified via the gray intensity analysis (normalized to β-actin). n = 3 independent experimental cell lines. The data are mean ± SD. Source data are provided as a Source Data file.

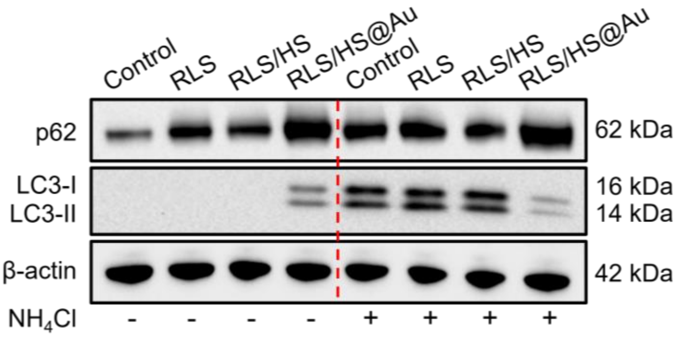

Supplementary Figure 23. Western blot analysis of autophagy-related proteins in HepG2 cells after treatment with various lipoplexes (at DNA of 1 μg/mL) in the absence or presence of autophagic flux inhibitor NH_4Cl (10 mmol/L) for 24 h. The β-actin was

used as a loading control.

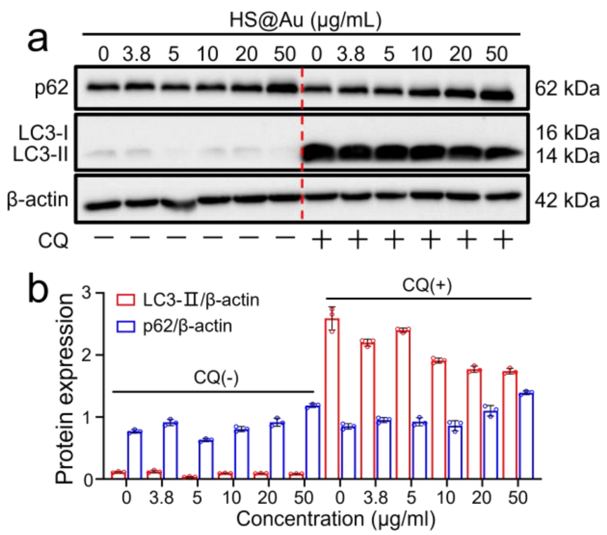

Supplementary Figure 24. **a** Western blot analysis of autophagy-related proteins in HepG2 cells treated with HS@Au at indicated concentrations in the absence or presence of autophagic flux inhibitor chloroquine (CQ, 10 μmol/L) for 24 h.β-actin was used as a loading control. **b** the expression levels of LC3-II and p62 quantified by gray intensity analysis (normalized to β-actin). n = 3 independent experimental cell lines. The data are mean ± SD. Source data are provided as a Source Data file.

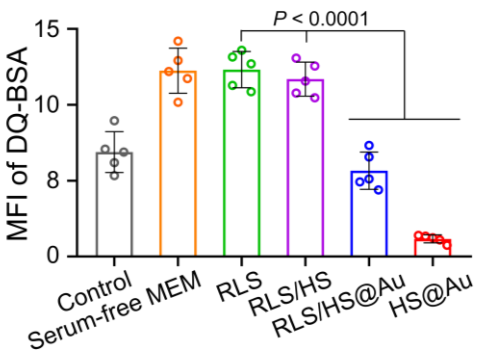

Supplementary Figure 25. The MFI of the brightly fluorescent fragments released by lysosomal degradation of DQ-BSA. The intensity was quantified by gray intensity analysis in ImageJ software. n = 5 fields from 3 independent experimental cell lines). Two-sided unpaired Student's t test was used for the comparisons. The data are mean ± SD. *p* values < 0.05 were considered statistically significant. Source data are provided as a Source Data file.

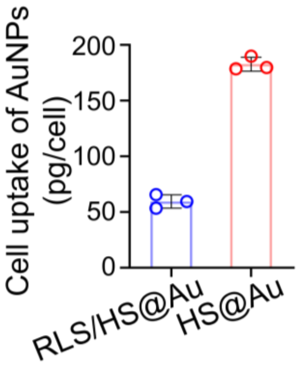

Supplementary Figure 26. Comparisons of intracellular concentration of gold after incubation with RLS/HS@Au, and HS@Au, respectively. n = 3 independent experimental cell lines. The data are mean ± SD. Source data are provided as a Source Data file.

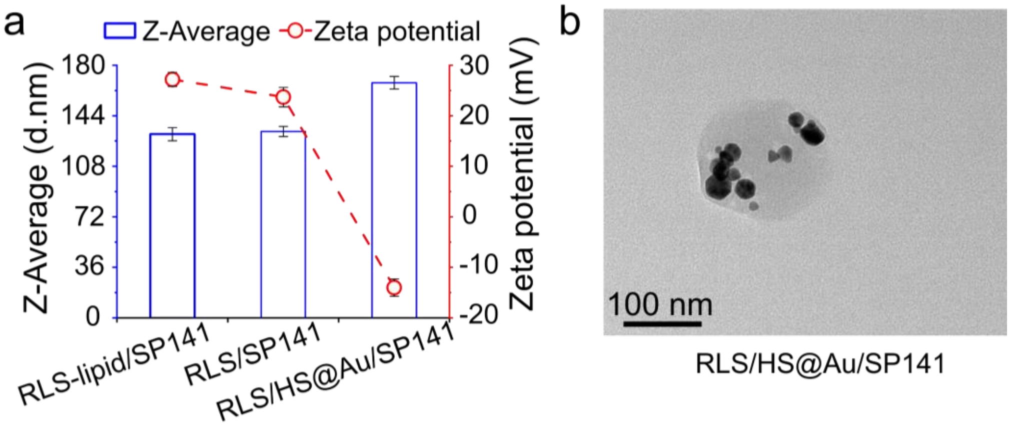

Supplementary Figure 27. Characterization of RLS/HS@Au/SP141 gene lipoplexes. a Size distribution and zeta potential of RLS-lipid/SP141, RLS/SP141, and RLS/HS@Au/SP141 gene lipoplexes. n = 3 independent experimental units. The data are mean ± SD. **b** The representative TEM image of RLS/HS@Au/SP141. n = 3 independent experimental units with similar results. The scale bar is 100 nm. Source data are provided as a Source Data file.

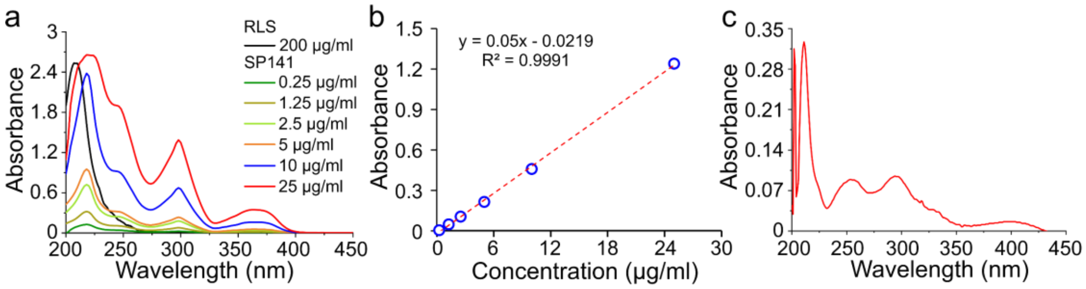

Supplementary Figure 28. The establishment of standard curves for SP141. a Absorption spectra of RLS and SP141 at different concentrations based on the ultraviolet spectrophotometer. **b** The standard curves of SP141. **c** Representative absorption spectrum of RLS/SP141 at weight ratio of 100:1. n = 3 independent experiments with similar results.

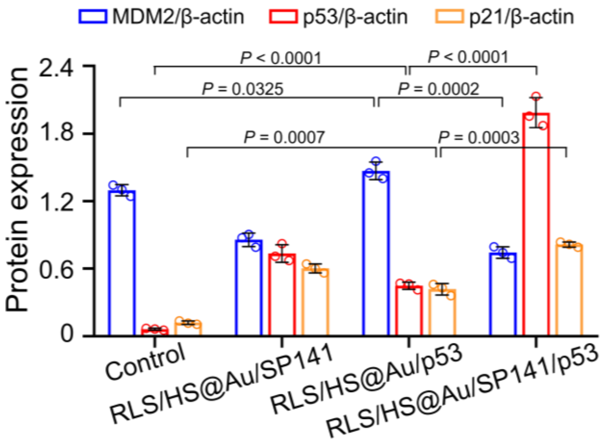

Supplementary Figure 29. Quantitate analysis of protein expression level of MDM2, p53, and p21 determined by western blot. n = 3 independent experimental cell lines. Two-sided unpaired Student's t test was used for the comparisons. The data are mean ± SD. *p* values < 0.05 were considered statistically significant Source data are provided as a Source Data file.

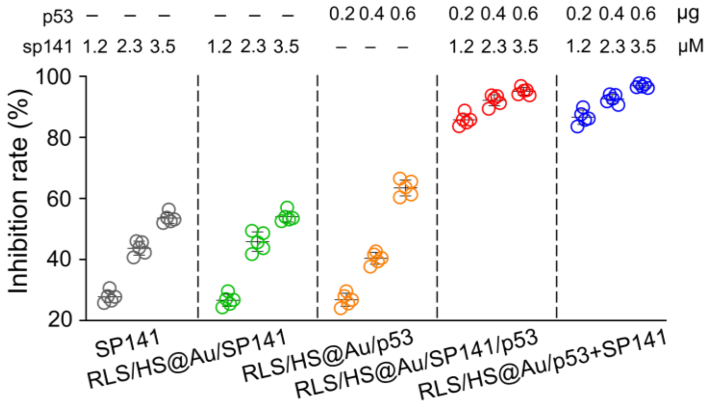

Supplementary Figure 30. The inhibition rate of various complexes on HepG2 cells measured by CCK-8 assay. The data are mean ± SD. n = 5 independent experimental cell lines. Source data are provided as a Source Data file.

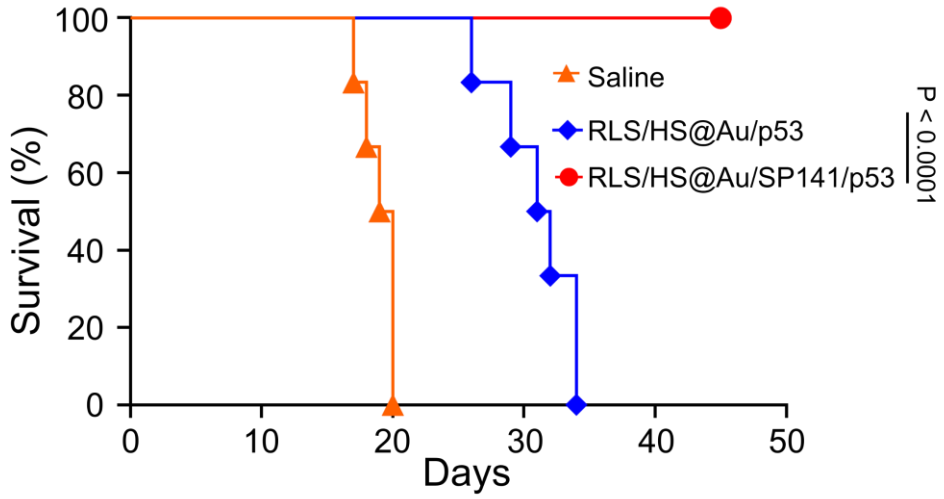

Supplementary Figure 31. Survival curves of HepG2 tumor-bearing Balb/c nude mice in 45 days after the treatments. Statistical significance was calculated by survival curve comparison with Log-rank (Mantel-Cox) test. p value < 0.05 was considered statistically significant. n = 6 mice. Source data are provided as a Source Data file.

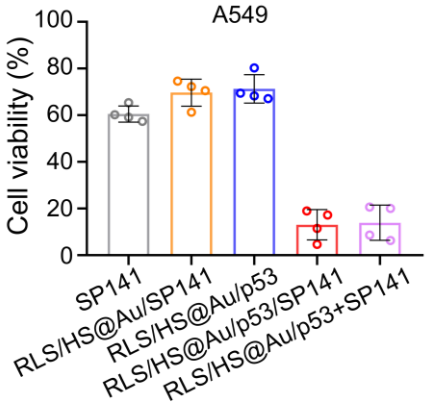

Supplementary Figure 32. The cytotoxicity of various lipoplexes on A549 cells measured by CCK-8 assay. The dosage of p53 plasmid and SP141 were 2 μg/mL and 1 μmol/L, respectively. The data are mean ± SD. n = 4 independent experimental cell lines. Source data are provided as a Source Data file.

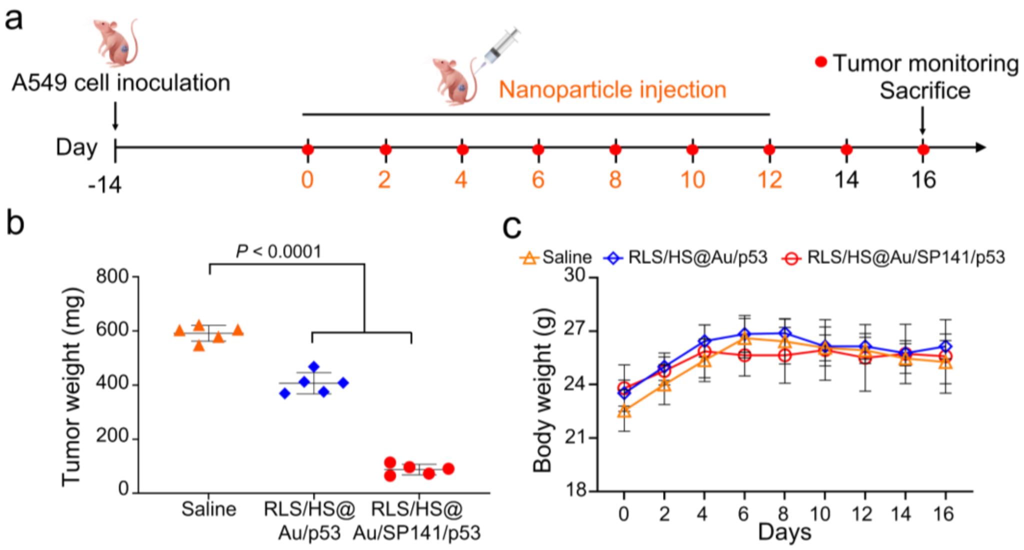

Supplementary Figure 33. In vivo antitumor efficacy of RLS/HS@Au/SP141/p53 against A549 tumor models. **a** Establishment of the subcutaneous A549 tumor model and dosing regimen. **b** Statistic analysis of the A549 tumor weight excised from different treatment groups. n = 5 mice. **c** Statistic analysis of body weight change of Balb/c nude mice with A549 xenografts in different groups. n = 5 mice. Two-sided unpaired Student's t test was used for the comparisons. The data are mean ± SD. *p* value < 0.05 was considered statistically significant. Source data are provided as a Source Data file.

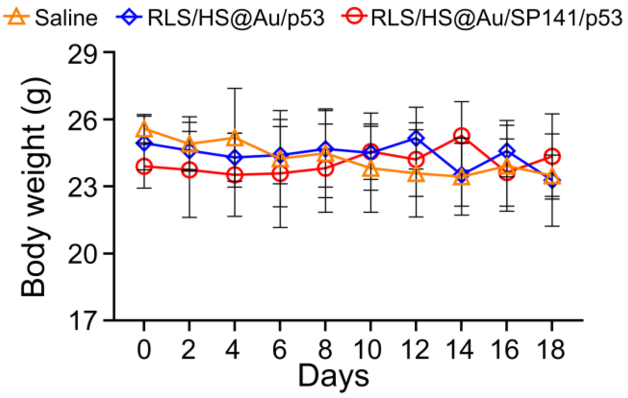

Supplementary Figure 34. Body weight change of Balb/c nude mice with HepG2 xenografts in different groups. n = 5 mice. The data are mean ± SD.

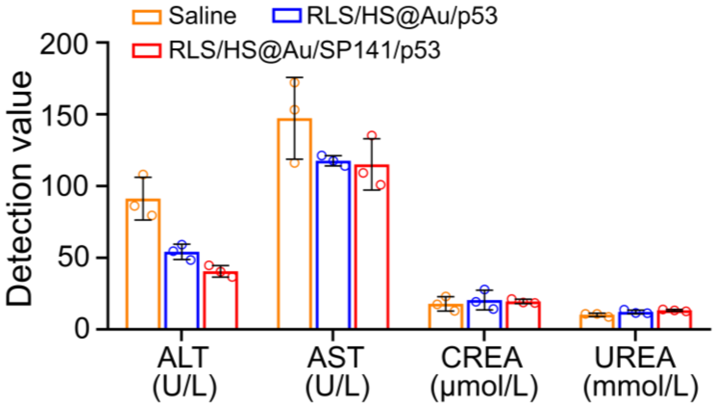

Supplementary Figure 35. Evaluation of the hepatic and renal functions of HepG2 tumor-bearing mice after different lipoplexes treatment. n = 3 mice. The data are mean ± SD.

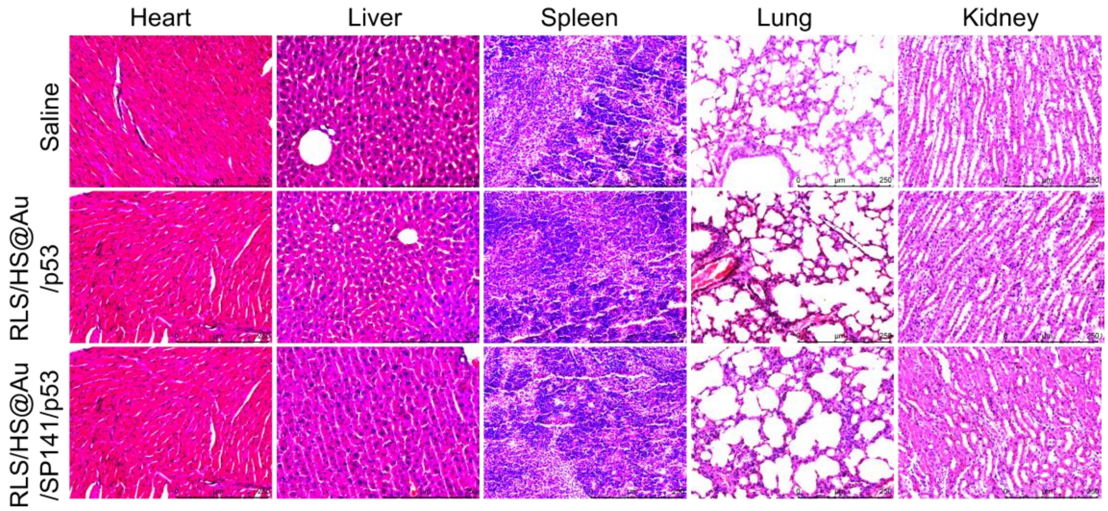

Supplementary Figure 36. Representative H&E staining images of main organs in

different groups on day 18. n = 3 mice from each experimental group. The scale bars are 250 μm.

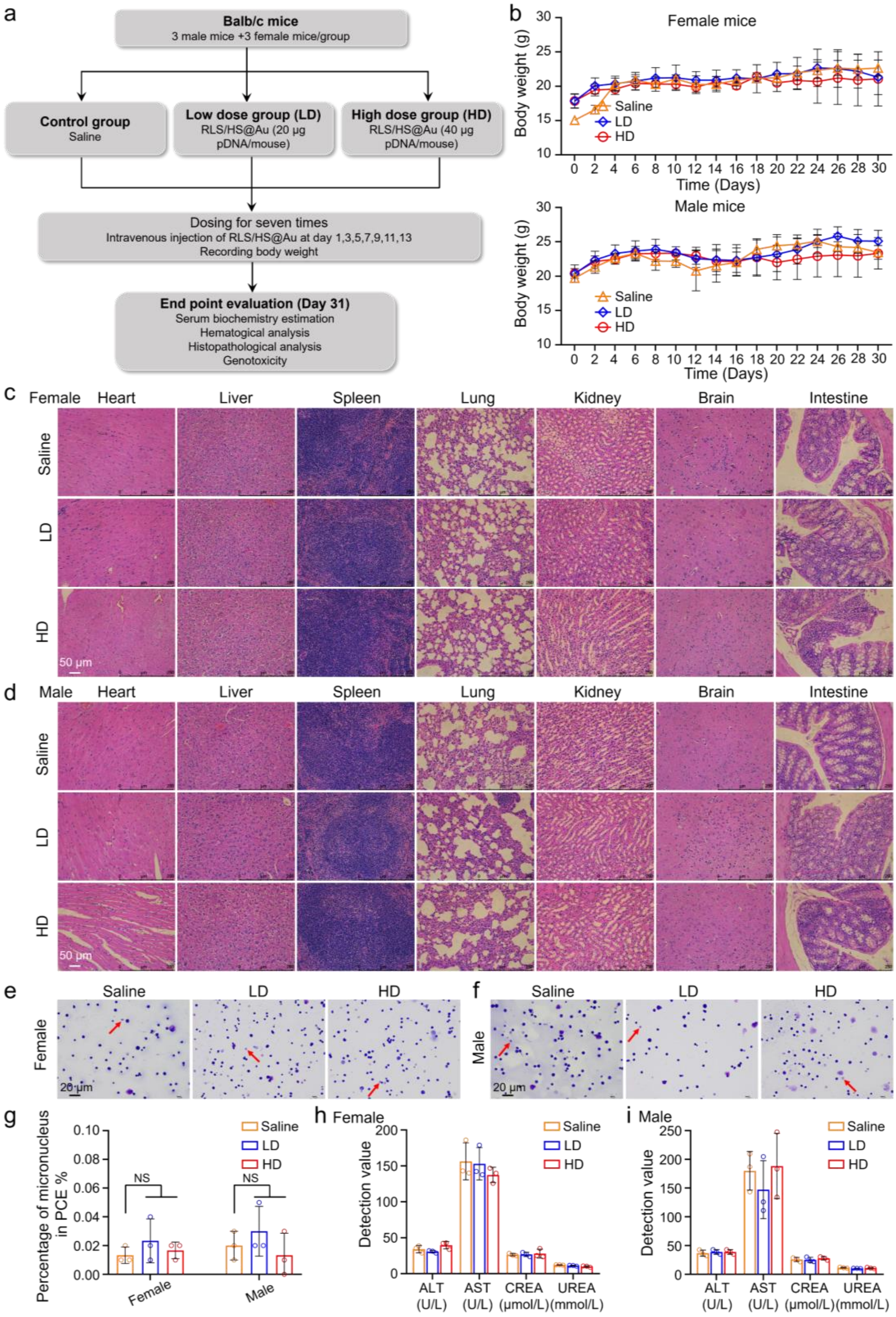

Supplementary Figure 37. Evaluation of the biosafety of hybrid lipoplexes in

Balb/c mice. a Schematic illustration of safety evaluation of RLS/HS@Au in mice. Balb/c mice were randomly divided into 3 groups, including saline-treatment control (Saline), low-dose (20 μg pDNA/mouse, LD), and high-dose (40 μg pDNA/mouse, HD) treated groups. Each group contained 3 female and 3 male mice. RLS/HS@Au/pDNA was intravenously injected seven times on days 1, 3, 5, 7, 9, 11, and 13. **b** Body weight changes of female and male mice throughout the study. n = 5 mice. Representative images of H&E stained organ sections isolated from female mice (**c**) and male mice (**d**) on day 31. n = 3 mice. The scale bar is 50 μm. **e**, **f**, and **g** Representative microscopic images of micronuclei formation from female mice (**e**) and male mice (**f**) on day 31, and corresponding quantification of the percentage of micronucleus (**g**) from 200 polychromatic erythrocytes (PCE) in each group. Red arrows represent the micronucleus formed in the PCE. n = 3 mice. The scale bar is 20 μm. **h**, **i** Blood biochemistry analysis of ALT, AST, CREA, and UREA in serum of female mice (**h**) and male mice (**i**) on day 31. n = 3 mice. Two-sided unpaired Student's t test was used for the comparisons in (**g**). The data in (**b**, **g**, **h**, and **i**) are mean ± SD. NS means no significance. Source data are provided as a Source Data file.

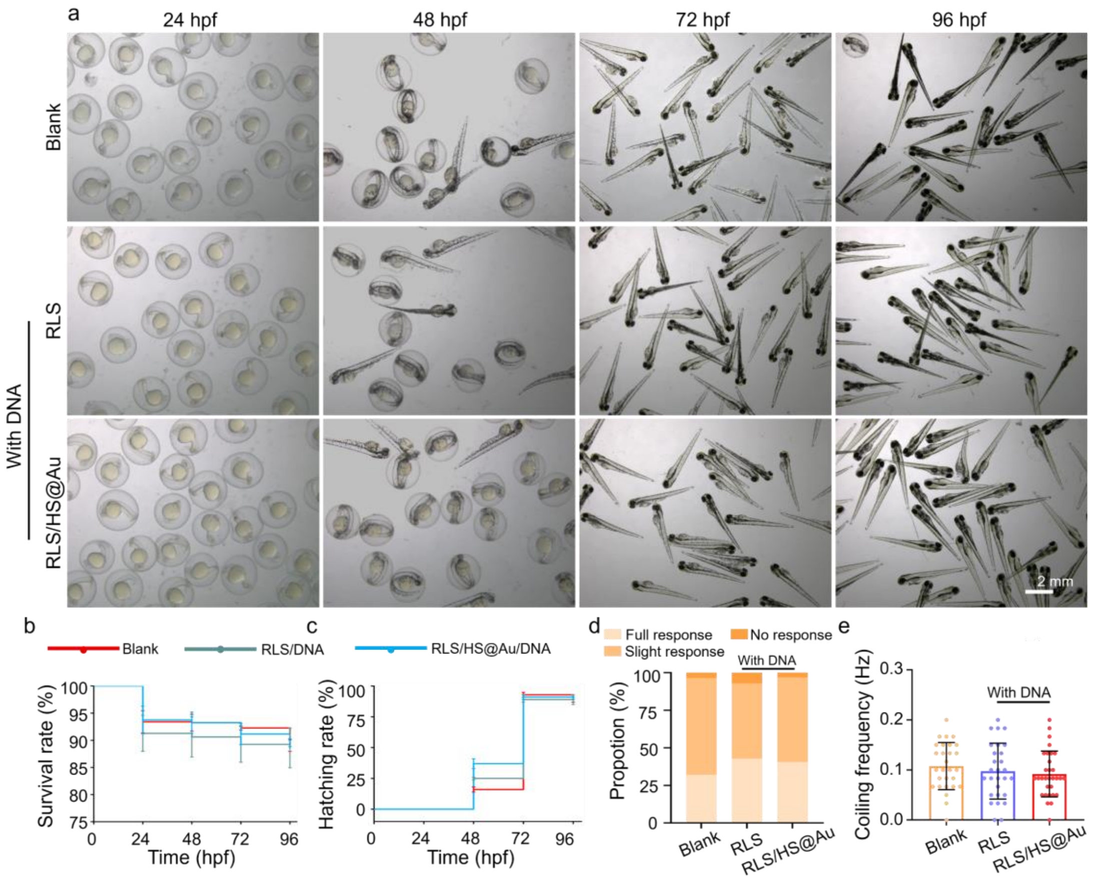

Supplementary Figure 38. Developmental toxicity and teratogenicity in zebrafish embryos. **a** Representative images of zebrafish embryos after injection with various lipoplexes at 48-, 72-, and 96-hours post-fertilization (hpf). **b** Survival rate and **c** hatching rate of zebrafish embryos at 24, 48, 72, and 96 hpf. n = 3 independent experiment. **d** the spontaneous contraction of the embryos examined at the 18-somite stage. Representative of 3 independent experimental zebrafish groups with similar results. **e** Total coiling contractions within 1 min counted at 24 hpf. n = 28-31 zebrafish per group. The data in (**b, c**, and **e**) are mean ± SD. Source data are provided as a Source Data file.

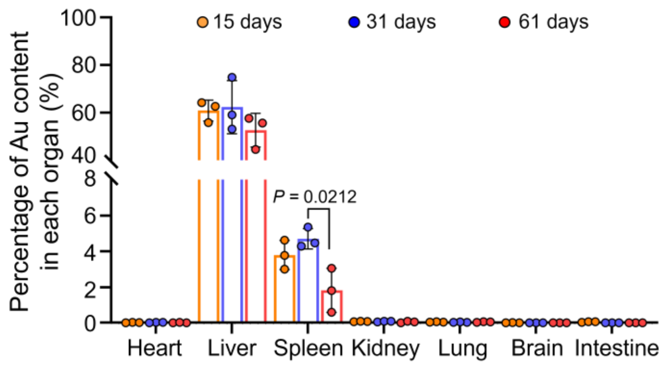

Supplementary Figure 39. Distribution of RLS/HS@Au in different organ tissues of Balb/c mice at 15, 31, and 61 days, expressed as the percentage of gold mass in the total injected dose. n = 3 mice. Two-sided unpaired Student's t test was used for the comparisons. The data are mean ± SD. p values < 0.05 were considered statistically significant. Source data are provided as a Source Data file.

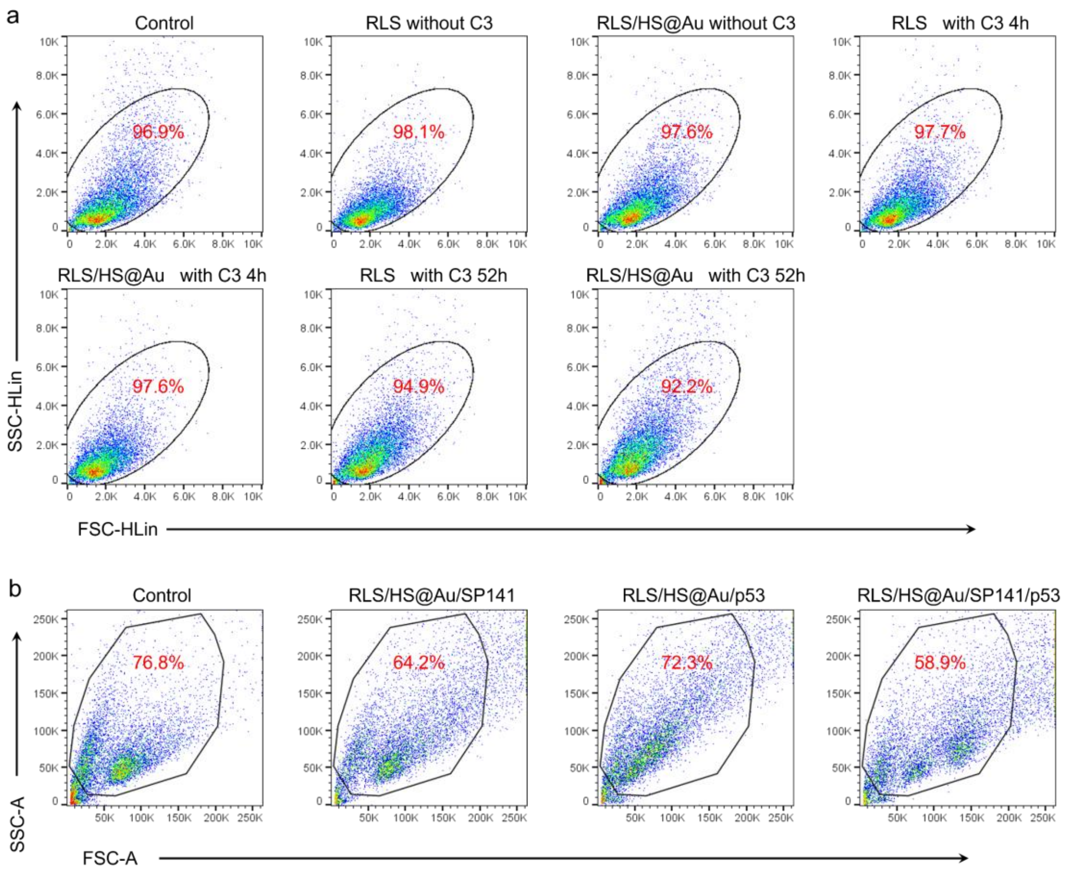

Supplementary Figure 40. Gating strategy for flow cytometric studies in Fig. 4d and Fig. 7e. a The initial gating involved the exclusion of debris with FSC/SSC for pEGFP transfection assay. **b** Apoptosis assays involve positive selection for live cells and subsequent gate selection to exclude negatively stained cells.

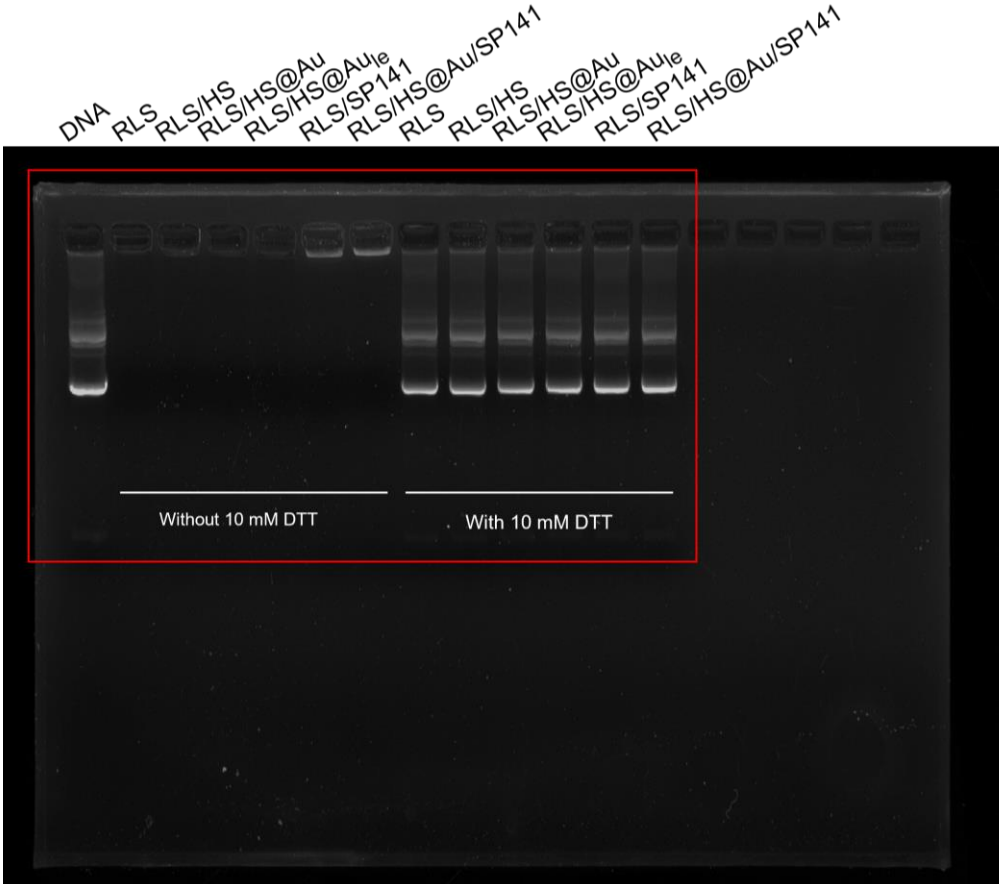

Supplementary Figure 41. Uncropped scan of gel presented in Supplementary Fig. 6.

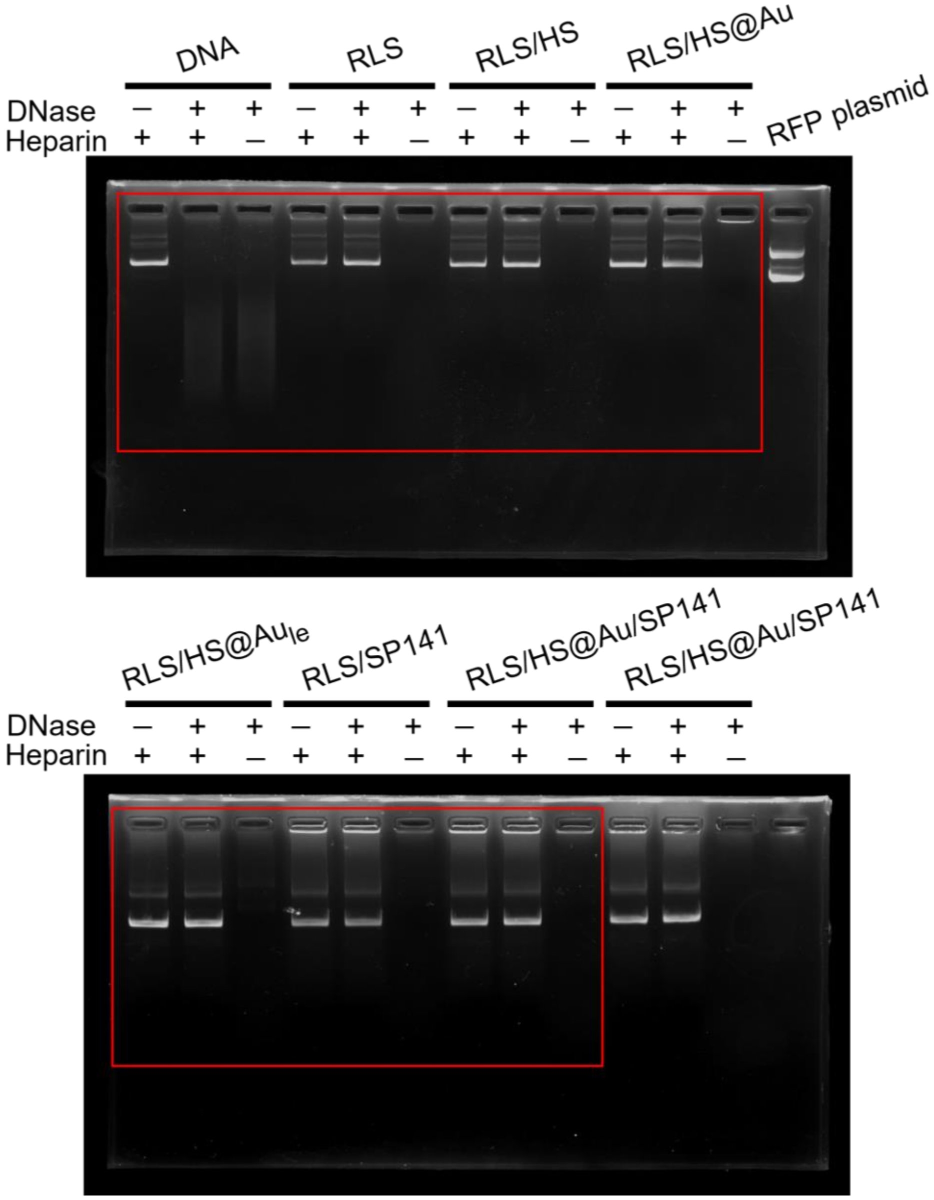

Supplementary Figure 42. Uncropped scans of gels presented in Supplementary Fig. 7.

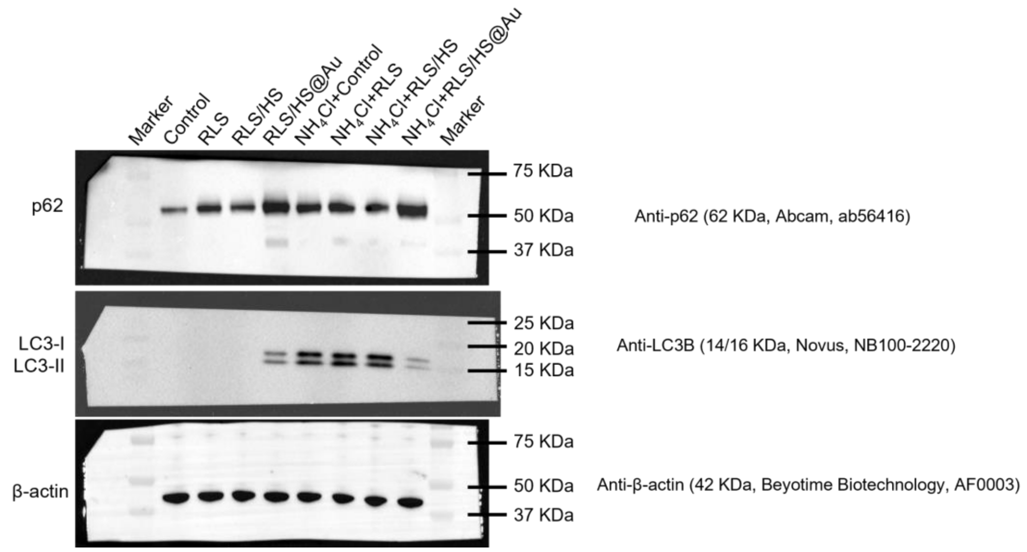

Supplementary Figure 43. Uncropped scans of blots presented in Supplementary Fig. 23.

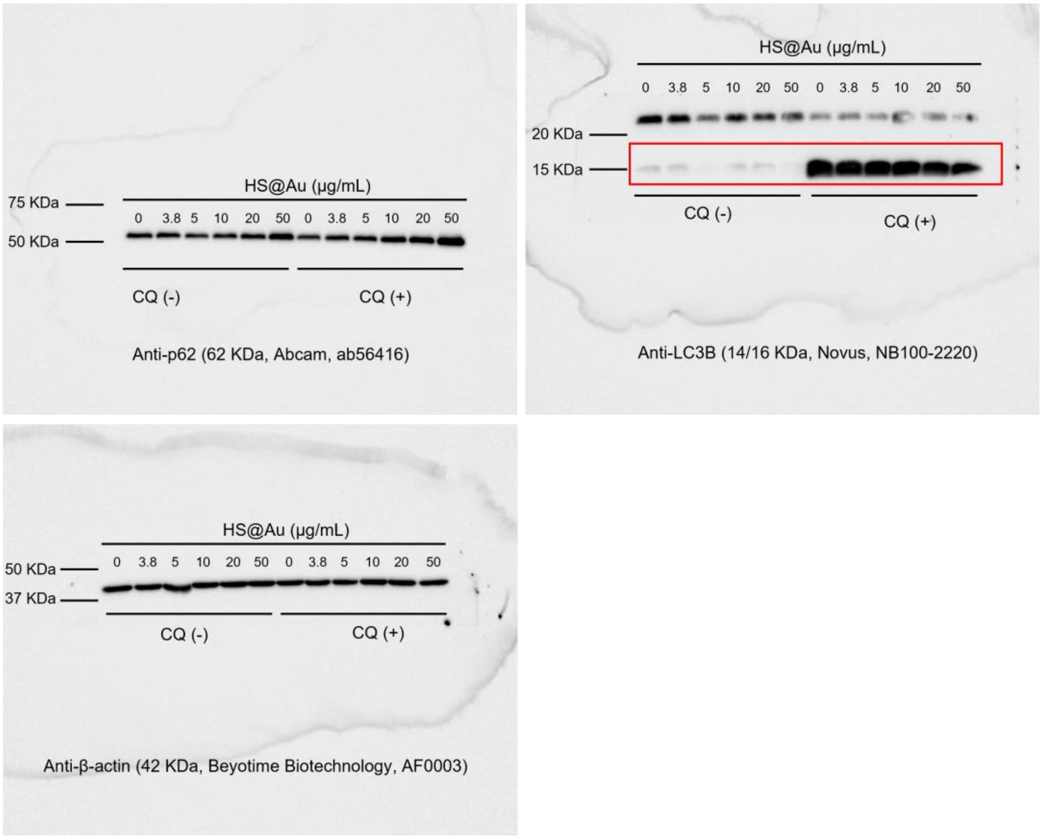

Supplementary Figure 44. Uncropped scans of blots presented in Supplementary Fig. 24.

Supplementary Table 1. The drug loading capacity (LC) and encapsulation efficiency (EE) of the lipoplexes.

Lipoplexes	LC (w/w, %)	EE (w/w, %)
RLS/SP141	0.95 ± 0.03	96.6 ± 0.8

LC (%) = drug loading capacities.

EE (%) = drug encapsulation efficiencies.

Supplementary Table 2. Hematological parameters of mice treated with RLS/HS@Au at the termination of the study.

Parameters	Groups (Female)			Groups (Male)			Reference value
	Control	LD	HD	Control	LD	HD	
WBC (× 10^9/L)	4.46 ± 1.05	4.72 ± 0.48	5.68 ± 2.54	4.43 ± 0.70	5.15 ± 1.05	7.45 ± 3.83	0.80 - 10.60
Neu%	23.10 ± 3.40	23.87 ± 2.31	22.7± 3.54	47.63 ± 7.00	32.03 ± 7.03	40.30 ± 3.89	6.5 - 50.0
Lym%	71.47 ± 4.63	69.57 ± 4.42	76.1 ±1.02	48.77 ± 6.50	63.97 ± 6.88	55.43 ± 5.40	40.0 - 92.0
Mon%	4.07 ± 1.43	2.20 ± 0.14	3.57 ± 1.60	2.70 ± 0.64	2.67 ± 0.31	3.1 ± 0.51	0.9 - 18.0
Eos%	1.37 ± 0.52	1.00 ± 0.42	0.97 ± 0.25	0.90 ± 0.22	1.33± 0.33	0.50 ± 0.08	0.0 - 7.5
Bas%	0.00 ± 0.00	0.03 ± 0.05	0.00 ±0.00	0.00 ± 0.00	0.00 ± 0.00	0.00 ± 0.00	0.0 – 1.5
RBC (× 10^{12}/L)	10.11 ± 0.07	10.64 ± 0.35	9.89 ± 0.75	11.37 ± 0.09	10.41 ± 0.86	11.15 ± 0.52	6.50 – 11.50
HGB (g/L)	161.67 ± 2.62	161.33 ± 0.47	162.67 ± 3.40	155.33 ± 3.86	168.00 ± 6.48	147.67 ± 4.11	110 – 165
HCT (%)	45.67 ± 0.46	47.63 ± 0.78	45.53 ± 2.36	49.87 ± 0.46	45.63 ± 3.83	47.13 ± 1.47	35.0 – 55.0
MCV (fL)	45.17 ± 0.69	44.83 ± 0.90	44.63 ± 0.97	43.87 ± 0.60	43.87 ± 0.52	42.33 ± 1.03	41.0 – 55.0
MCH (pg)	16.00 ± 0.36	15.87 ± 0.37	15.53 ± 0.41	15.43 ± 0.31	15.53 ± 0.21	15.10 ± 0.37	13.0 – 18.0
MCHC (g/L)	353.67 ± 5.25	353.67 ± 0.94	348.33 ± 2.62	352.00 ± 6.16	353.67 ± 2.05	356.00 ± 2.83	300 – 360
RDW-CV (%)	15.10 ± 0.36	15.27 ± 0.17	16.17 ± 1.31	15.23 ± 0.42	15.93 ± 0.83	16.40 ± 0.62	12.0 – 19.0
RDW-SD (%)	30.70 ± 0.37	30.53 ± 0.71	32.27 ± 1.96	29.93 ± 1.36	31.23 ± 1.27	31.37 ± 0.45	23.0 – 39.0
PLT (× 10^{11}/L)	10.49 ± 0.36	10.88 ± 0.80	10.68 ± 1.49	10.10 ± 0.53	10.88 ± 1.49	9.11 ± 1.04	4.00 – 16.00
MPV (fL)	6.20 ± 0.16	6.00 ± 0.08	6.03 ± 0.25	6.07 ± 0.12	5.93 ± 0.12	5.97 ± 0.17	4.0 – 6.2
PDW (fL)	16.63 ± 0.12	16.33 ± 0.09	16.30 ± 0.22	16.40 ± 0.22	16.33 ± 0.19	16.33 ± 0.26	12.0 – 17.5
PCT (%)	0.65 ± 0.01	0.65 ± 0.04	0.64 ± 0.07	0.64 ± 0.01	0.65 ± 0.10	0.54 ± 0.07	0.100 – 0.780

WBC, White blood cell counts; Neu, Neutrophils; Lym, Lymphocytes; Mon, Monocytes; Eos, Eosinophils; Bas, Basophils; RBC, Red blood cell counts; HGB, Hemoglobin; HCT, Hematocrit; MCV, Mean corpuscular volume; MCH, Mean corpuscular hemoglobin; MCHC, Mean corpuscular hemoglobin concentration; RDW-CV, Red cell distribution width-coefficient variation; RDW-SD, Red cell distribution width-standard deviation; PLT, Platelets; MPV, Mean platelet volume; PDW, Platelet distribution width; PCT, Plateletcrit. n = 3 mice. The data are mean ± SD. Source data are provided as a Source Data file.

Supplementary Table 3. Malformation rate induced by various lipoplexes in zebrafish larvae.

Name	Malformation of tail	Scoliosis	Pericardial edema
Blank	0	1.29% ± 0.04	1.29% ± 0.04
RLS	1.63% ± 0.13	0	0
RLS/HS@Au	0	0	0
