# Supplementary material for: The hybrid lipoplex induces cytoskeletal rearrangement via autophagy/RhoA signaling pathway for enhanced anticancer gene therapy
Source: Nat Commun. 2025 Jan 2;16:339. doi: 10.1038/s41467-024-55727-4 (PMC11696071; doi:10.1038/s41467-024-55727-4)
Supplement: Supplementary file 3 — Reporting Summary [file 41467_2024_55727_MOESM3_ESM.pdf]

nature portfolio

Corresponding author(s): Rongrong Jin, Yu Nie

Last updated by author(s): Dec 2, 2024

Reporting Summary

Nature Portfolio wishes to improve the reproducibility of the work that we publish. This form provides structure for consistency and transparency in reporting. For further information on Nature Portfolio policies, see our Editorial Policies and the Editorial Policy Checklist.

Statistics

For all statistical analyses, confirm that the following items are present in the figure legend, table legend, main text, or Methods section.

n/a	Confirmed	
☐	☒	The exact sample size (*n*) for each experimental group/condition, given as a discrete number and unit of measurement
☐	☒	A statement on whether measurements were taken from distinct samples or whether the same sample was measured repeatedly
☐	☒	The statistical test(s) used AND whether they are one- or two-sided<br>*Only common tests should be described solely by name; describe more complex techniques in the Methods section.*
☒	☐	A description of all covariates tested
☒	☐	A description of any assumptions or corrections, such as tests of normality and adjustment for multiple comparisons
☐	☒	A full description of the statistical parameters including central tendency (e.g. means) or other basic estimates (e.g. regression coefficient) AND variation (e.g. standard deviation) or associated estimates of uncertainty (e.g. confidence intervals)
☐	☒	For null hypothesis testing, the test statistic (e.g. *F*, *t*, *r*) with confidence intervals, effect sizes, degrees of freedom and *P* value noted<br>*Give P values as exact values whenever suitable.*
☒	☐	For Bayesian analysis, information on the choice of priors and Markov chain Monte Carlo settings
☒	☐	For hierarchical and complex designs, identification of the appropriate level for tests and full reporting of outcomes
☐	☒	Estimates of effect sizes (e.g. Cohen's *d*, Pearson's *r*), indicating how they were calculated

Our web collection on statistics for biologists contains articles on many of the points above.

Software and code

Policy information about availability of computer code

Data collection

Data in Figs. 2b, 2d, Supplementary Fig. 3b, Supplementary Fig. 8, Supplementary Fig. 9 and Supplementary Fig. 27a were characterized by dynamic light scattering (DLS) using a Zetasizer Nano Zs (Malvern Instruments, UK).
Data in Figs. 2c, Supplementary Fig. 3c and Supplementary Fig. 27b were observed by transmission electron microscopy (TEM, JEM-1011, Japan).
Data in Supplementary Fig. 2b were obtained by gel permeation chromatography (GPC, Waters 1515, USA).
Data in Figs. 4g, Supplementary Fig. 20 were observed by transmission electron microscopy (TEM, JEM-1400-FLASH, Japan).
Data in Figs. 2e, f were obtained by atomic force microscopy (AFM, Multimode 8, Bruker).
Data in Figs. 2h was obtained by the live-cell imaging system (DMI6000B, Leica), and analyzed by ImageJ software (Fiji, Ver. 2.14.0).
Data in Figs. 2g, 3c, 3e, 4b, 4f, 4i, 5a, 5c, 7c, 8e (TUNEL assay), Supplementary Fig. 10a and Supplementary Fig. 19a were observed by a confocal laser scanning microscope (CLSM, LSM 880, Zeiss).
Data in Figs. 5e (tumor penetration images of lipoplexes) was observed by CLSM (Nikon, A1R MP+).
Data in Figs. 3a, 4c, 8g, Supplementary Fig. 11a, Supplementary Fig. 11c, Supplementary Fig. 11e, Supplementary Fig. 12a, Supplementary Fig. 12c and Supplementary Fig. 12e were observed by an inverted fluorescence microscope (Olympus, Japan).
Data in Figs. 3g, 3h were obtained by ZEN 2.3 (Blue edition, Zeiss), followed by the 3D-construction with Imaris 9.0.1 software, and analyzing the corresponding cell CLSM image.
Data in Figs. 4d and 7e were conducted using the flow cytometer (BD Accuri C6, USA).
Data in Figs. 4a, 4h, 7b, 8f, Supplementary Fig. 6, Supplementary Fig. 7, Supplementary Fig. 23 and Supplementary Fig. 24a were observed by the Molecular Imager ChemiDoc XRS+ (Bio-Rad, USA).
Data in Figs. 6a-6d were used IVIS Lumina Series III imaging system (Perkin Elmer, USA).
Data in Figs. 7a, Supplementary Fig. 4, Supplementary Fig. 28 and Supplementary Table 1 were measured by UV-visible spectrophotometer (UV 2600, Japan).

Data in Fig. 7d, Supplementary Fig. 13, Supplementary Fig. 14, Supplementary Fig. 30 and Supplementary Fig. 32 were obtained by microplate reader (Bio-Rad 550,USA).
Data in Fig. 8e (H&E, Immunohistology), Supplementary Fig. 36 and Supplementary Fig. 37c, d were observed by an upright microscope (DM1000, Leica).
Data in Fig. 5e (overall tumor morphology), 8c, 8i and Supplementary figure 38a were observed by stereomicroscopy (Carl zeiss, SteREO Discovery V20).
Data in Supplementary figure 37e, f were observed by digital camera microscope (BA210Digital).
All softwares used in this study have been described in detail in methods part.

Data analysis

Chemical structures were drawn by ChemDraw 21. The MestReNova Version 10.0.1 was used for 1H NMR data analysis. Young's modulus was additionally determined through NanoScope Analysis software (Ver. 1.5) in atomic force microscopy (AFM, Multimode 8, Bruker) . The CLSM images were analyzed by the ImageJ software (Fiji, Ver. 2.14.0). The results of the flow cytometer were analyzed by FlowJo software (Ver. 10). In vivo bioluminescent imaging data were analyzed by the IVIS Spectrum Software (Ver. 4.4). Origin 8.0 and GraphPad Prism 9.0 were used for other statistical analyses. The results are reported as the mean ± SD (standard deviation). p values < 0.05 were considered statistically significant.
All softwares used in this study have been described in detail in the methods part.

For manuscripts utilizing custom algorithms or software that are central to the research but not yet described in published literature, software must be made available to editors and reviewers. We strongly encourage code deposition in a community repository (e.g. GitHub). See the Nature Portfolio guidelines for submitting code & software for further information.

Data

Policy information about availability of data

All manuscripts must include a data availability statement. This statement should provide the following information, where applicable:

- Accession codes, unique identifiers, or web links for publicly available datasets
- A description of any restrictions on data availability
- For clinical datasets or third party data, please ensure that the statement adheres to our policy

All data supporting the findings of this study are available within the article and its supplementary files. Any additional requests for information can be directed to, and will be fulfilled by, the corresponding author. Source data are provided with this paper.

Research involving human participants, their data, or biological material

Policy information about studies with human participants or human data. See also policy information about sex, gender (identity/presentation), and sexual orientation and race, ethnicity and racism.

Reporting on sex and gender	N/A
Reporting on race, ethnicity, or other socially relevant groupings	N/A
Population characteristics	N/A
Recruitment	N/A
Ethics oversight	N/A

Note that full information on the approval of the study protocol must also be provided in the manuscript.

Field-specific reporting

Please select the one below that is the best fit for your research. If you are not sure, read the appropriate sections before making your selection.

☒ Life sciences ☐ Behavioural & social sciences ☐ Ecological, evolutionary & environmental sciences

For a reference copy of the document with all sections, see nature.com/documents/nr-reporting-summary-flat.pdf

Life sciences study design

All studies must disclose on these points even when the disclosure is negative.

Sample size

No statistical methods were used to predetermine the sample size. The sample size was selected empirically following previous experience in the assessment of experimental variability. For physicochemical experiments related to the characterization of materials and in vitro cell experiments, n=3 was chosen as the minimal replicate number. For all in vivo studies, at least 3 random animals were selected for statistical analysis in each experiment. The "n" number in the article represents several definitions, including biologically independent samples, independent experiments, and cells examined over "n " independent experiments. The number of biological replicates is also reported in the relevant figure legends in the manuscript. In addition, we adhere to sample size requirements necessary for determining statistical significance.

Data exclusions	No data were excluded.
Replication	All experiments were performed in "n" independent replicates. Experimental repeat numbers are also reported in figure legends.
Randomization	All samples were randomly allocated into experimental groups.
Blinding	No formal blinding was used in this study. Because all experiments were performed based on standardized protocols and blinding has no effect on the experiment results. Unbiased experimental procedure and data analysis were carried out as far as possible.

Reporting for specific materials, systems and methods

We require information from authors about some types of materials, experimental systems and methods used in many studies. Here, indicate whether each material, system or method listed is relevant to your study. If you are not sure if a list item applies to your research, read the appropriate section before selecting a response.

Materials & experimental systems

n/a	Involved in the study	
☐	☒	Antibodies
☐	☒	Eukaryotic cell lines
☒	☐	Palaeontology and archaeology
☐	☒	Animals and other organisms
☒	☐	Clinical data
☒	☐	Dual use research of concern
☒	☐	Plants

Methods

n/a	Involved in the study	
☒	☐	ChIP-seq
☐	☒	Flow cytometry
☒	☐	MRI-based neuroimaging

Antibodies

Antibodies used

All the antibodies were provided in the method section.
1)Mouse anti-RhoA, catalog number: ARH05; clone number: 64D6.1.16; lot number: 003 (1:400 dilution);
2) Mouse anti-p62, catalog number: ab56416; clone number: 2C11; lot number: GR3374761-1 (1:1000 dilution);
3) Rabbit anti-LC3, catalog number: NB100-2220; clone number: N/A; lot number: D155067 (1:1000 dilution);
4) Rabbit anti-LAMP1, catalog number: 9091T; clone number: D2D11; lot number: 8 (1:400 dilution);
5) Mouse anti-MDM2, catalog number: sc-965; clone number: SMP-14; lot number: J2922 (1:1000 dilution);
6) Mouse anti-p21, catalog number: sc-817; clone number: 187; lot number: E2620 (1:1000 dilution);
7) Mouse anti-β-actin, catalog number: AF0003; clone number: N/A; lot number: 102723240430 (1:1000 dilution);
8) Mouse anti-p53, catalog number: sc-126; clone number: DO-1; lot number: I2722 (1:1000 dilution);
9) Goat anti-mouse IgG-HRP antibody, catalog number: 7076P2; clone number: N/A; lot number: 36 (1:5000 dilution);
10) Goat anti-rabbit IgG-HRP antibody, catalog number: 7074P2; clone number: N/A; lot number: 32 (1:5000 dilution);
11) Goat anti-mouse IgG (H+L) AlexaFluor-488 conjugated antibody, catalog number: 4408S; clone number: N/A; lot number: 22 (1:2000 dilution);
12) Goat anti-rabbit IgG (H+L) AlexaFluor-647 conjugated antibody, catalog number: 4414S; clone number: N/A; lot number: 26 (1:2000 dilution).

Validation

RhoA: https://www.cytoskeleton.com/arh05
p62: https://www.abcam.cn/products/primary-antibodies/sqstm1--p62-antibody-2c11-bsa-and-azide-free-ab56416.html
LC3: https://www.novusbio.com/products/lc3b-antibody_nb100-2220
LAMP1: https://www.cellsignal.cn/products/primary-antibodies/lamp1-d2d11-xp-rabbit-mab/9091
MDM2: https://www.scbt.com/zh/p/mdm2-antibody-smp14
p21: https://www.scbt.com/zh/p/p21-antibody-187
β-actin: https://www.beyotime.com/product/AF0003.htm
p53: https://www.scbt.com/zh/p/p53-antibody-do-1
anti-mouse IgG-HRP: https://www.cellsignal.cn/products/secondary-antibodies/anti-mouse-igg-hrp-linked-antibody/7076
anti-rabbit IgG-HRP: https://www.cellsignal.cn/products/secondary-antibodies/anti-rabbit-igg-hrp-linked-antibody/7074
anti-mouse IgG (H+L) AlexaFluor-488: https://www.cellsignal.cn/products/secondary-antibodies/anti-mouse-igg-h-l-f-ab-2-fragment-alexa-fluor-488-conjugate/4408
Goat anti-rabbit IgG (H+L) AlexaFluor-647: https://www.cellsignal.cn/products/secondary-antibodies/anti-rabbit-igg-h-l-f-ab-2-fragment-alexa-fluor-647-conjugate/4414

Eukaryotic cell lines

Policy information about cell lines and Sex and Gender in Research

Cell line source(s)

The human hepatoma cell lines (HepG2, Cat. No: SCSP-510), lung carcinoma cell lines (A549, Cat. No: SCSP-503), human cervical carcinoma cell lines (Hela, Cat. No: SCSP-504), breast tumor cell lines (4T1, Cat. No: TCM32), and fibroblast cell lines (NIH-3T3, Cat. No: SCSP-515) were obtained from the Chinese Academy of science Cell Bank for Type Culture collection. (China).

Authentication	All cell lines were authenticated using STR analysis, and the last authentication testing time of HepG2 is 19/5/2024, A549 is 30/5/2023, Hela is 28/9/2023, 4T1 is 13/11/2023, NIH-3T3 is 15/8/2023.
Mycoplasma contamination	All cell lines tested negative for mycoplasma contamination by qPCR-based assay examination.
Commonly misidentified lines (See ICLAC register)	No commonly misidentified lines were used in the study.

Animals and other research organisms

Policy information about studies involving animals; ARRIVE guidelines recommended for reporting animal research, and Sex and Gender in Research

Laboratory animals	Male Balb/c nude mice (4-6 weeks) were purchased from GemPharmatech LLc. (Chengdu, china). Male and female Balb/c mice (4-6 weeks) were purchased from Dossy Experimental Animals Co., Ltd. (Chengdu, China). All mice were kept in an SPF environment. All mice were housed under a 12-hour light/12-hour dark cycle in an animal facility under specific pathogen-free conditions, and maintained at 25 °C with humidity levels between 40% to 70%.
Wild animals	Wild animals were not used in this study.
Reporting on sex	Separate studies have concluded there are no discernible differences between the sexes undergoing these treatments. Therefore, in order to maintain our commitment to the 3R's (replace, reduce, and refine) to reduce NHP (non-human primates) use, we did not include female animals in the treatment studies, which would have required additional animals to have parity between sexes in each group. We have supplemented safety assessments on both female and male mice, observing no significant differences. Therefore, sex is not a consideration in this study and these results could be applied to both sexes.
Field-collected samples	The study did not involve samples collected from that field.
Ethics oversight	All animal procedures were performed with ethical compliance and approval by the institutional Animal Care and Ethics Committee of Sichuan University (ethical approval code SCU46-2401-01)

Note that full information on the approval of the study protocol must also be provided in the manuscript.

Plants

Seed stocks	N/A
Novel plant genotypes	N/A
Authentication	N/A

Flow Cytometry

Plots

Confirm that:

☒ The axis labels state the marker and fluorochrome used (e.g. CD4-FITC).

☒ The axis scales are clearly visible. Include numbers along axes only for bottom left plot of group (a 'group' is an analysis of identical markers).

☒ All plots are contour plots with outliers or pseudocolor plots.

☒ A numerical value for number of cells or percentage (with statistics) is provided.

Methodology

Sample preparation	(1) For the in vitro pEGFP transfection analysis, HepG2 cells were collected after treatment, washed with PBS, and analyzed by fluorescent microscopy.<br>(2) For apoptosis assay, HepG2 cells were collected after treatment and washed with PBS. Apoptotic cells were detected by flow cytometer according to the Annexin V-FITC kit manufacturer's protocol.
Instrument	Flow cytometer (BD Accuri C6, USA)
Software	The cells were analyzed by FlowJo software (FlowJo Ver 10).

Cell population abundance	HepG2 cells with a homogeneous population were employed in this study. Therefore, 100000 cells per tube were collected for the analysis of pEGFP expression and HepG2 cell apoptosis.
Gating strategy	For all experiments, FSC-A/SSC-A gates of the starting cell population were used to discriminate between viable cells and cell debris, followed by positive selection for live cells, with a subsequent gate applied to select target cells while excluding other cells.

☒ Tick this box to confirm that a figure exemplifying the gating strategy is provided in the Supplementary Information.
